# Peripheral Vasopressor Use in Early Sepsis-Induced Hypotension

**DOI:** 10.1001/jamanetworkopen.2025.29148

**Published:** 2025-08-27

**Authors:** Elizabeth S. Munroe, Ivan N. Co, Ivor Douglas, Robert Hyzy, Akram Khan, Kristine Nelson, Pauline K. Park, Ithan D. Peltan, Todd W. Rice, Sarah Seelye, Wesley H. Self, Nathan I. Shapiro, Hallie C. Prescott

**Affiliations:** 1 Division of Pulmonary and Critical Care Medicine, Department of Medicine, University of Michigan, Ann Arbor; 2 Department of Emergency Medicine, University of Michigan, Ann Arbor; 3 Division of Pulmonary Sciences & Critical Care, Denver Health Medical Center, Denver, Colorado; 4 Division of Pulmonary and Critical Care Medicine, Department of Medicine, Oregon Health and Science University, Portland; 5 Department of Surgery, University of Michigan, Ann Arbor; 6 Division of Pulmonary and Critical Care Medicine, Intermountain Health, Murray, Utah; 7 Division of Allergy, Pulmonary, and Critical Care Medicine, Vanderbilt University, Nashville, Tennessee; 8 VA Center for Clinical Management Research, Ann Arbor, Michigan; 9 Department of Emergency Medicine, Vanderbilt University Medical Center, Nashville, Tennessee; 10 Department of Emergency Medicine, Beth Israel Deaconess Medical Center and Harvard Medical School, Boston, Massachusetts

## Abstract

**Importance:**

Evidence supporting the safety of infusing vasopressors through peripheral intravenous catheters (peripheral vasopressors) is largely derived from single-center studies, limiting generalizability.

**Objective:**

To evaluate factors associated with vasopressor route selection and assess safety and clinical outcomes of peripheral vasopressor administration in early sepsis resuscitation.

**Design, Setting, and Participants:**

This prospective cohort study is a secondary analysis of the Crystalloid Liberal vs Early Vasopressors in Sepsis (CLOVERS) trial conducted in 60 US hospitals from March 2018 to February 2022. Patients in CLOVERS who received vasopressors within 24 hours of enrollment and did not have central venous access at enrollment were included. Data were analyzed from January 2023 to June 2025.

**Exposure:**

Route of vasopressor initiation (central or peripheral).

**Main Outcomes and Measures:**

The primary analysis evaluated the route of vasopressor initiation, while the secondary analysis assessed continuation of peripheral vasopressors beyond 6 hours. Univariable and multivariable analyses of factors associated with vasopressor route were conducted, as was a multivariable analysis to evaluate the association of route with outcomes, including 90-day mortality. Descriptive statistics were used to summarize 28-day peripheral vasopressor and central venous catheter (CVC) complications.

**Results:**

Of 1563 patients in CLOVERS, 582 (37.2%) received vasopressors and met study inclusion criteria. Included patients had a median (IQR) age of 63 (52-72) years, and 267 (45.9%) were female, 96 (16.5%) were African American, 416 (71.5%) were White, and 70 (12.0%) were another race or had unreported race. Vasopressors were initiated via peripheral catheter in 490 patients (84.2%) and via central venous access in 92 patients (15.8%). Study site was the only factor independently associated with route of initiation (median odds ratio, 3.48; 95% CI, 1.57-5.38). In adjusted analyses, peripheral vs central initiation was associated with statistically comparable 90-day mortality (128 participants [26.1%] vs 34 participants [37.0%]; adjusted odds ratio, 0.67; 95% CI, 0.39-1.16). Peripheral vasopressors were continued beyond 6 hours in 333 of 490 patients (68.0%). Peripheral vasopressor complications were rare and low-grade (3 of 490 patients [0.6%]), with no cases of ulceration or tissue injury. In contrast, there were 14 complications from CVC placement occurring in 12 of 322 patients (3.7%) who had CVCs placed in the first 72 hours.

**Conclusions and Relevance:**

In this prospective cohort study of the CLOVERS trial, peripheral administration of vasopressors was common and was associated with low complication rates. These findings support the safety and feasibility of short-term peripheral vasopressor use in early sepsis resuscitation.

## Key Points

**Question:**

How are peripheral vasopressors used in early sepsis resuscitation, and is their use safe?

**Findings:**

In this cohort study of 582 patients from the Crystalloid Liberal vs Early Vasopressors in Sepsis trial, most patients had vasopressors initiated through peripheral venous catheters, and more than one-half had vasopressors continued through peripheral catheters beyond 6 hours. Complications of peripheral vasopressor administration were rare, and there was no association of vasopressor route with mortality, even after adjustment for patient characteristics.

**Meaning:**

These findings suggest that early peripheral vasopressor use is a feasible and safe approach for managing sepsis-induced hypotension in appropriately monitored settings.

## Teaser

This cohort study examines factors associated with central vs peripheral vasopressor route selection and assesses safety and clinical outcomes of peripheral vasopressor administration in early sepsis resuscitation.

## Introduction

Vasopressors are commonly used to treat sepsis-induced hypotension and shock.^1^ Historically, vasopressors have been administered through central venous catheters (CVCs) due to case reports of catastrophic tissue injury resulting from extravasation of vasopressors from peripheral intravenous catheters (PIVs).^2,3,4^ However, over the past decade, several studies have suggested that administering vasopressors through peripheral access—peripheral vasopressors—is associated with low rates of extravasation and virtually no instances of tissue injury.^5,6,7,8^ Peripheral vasopressors have practical advantages, including faster vasopressor initiation and avoidance of CVCs and their associated risks.^9^ These advantages and emerging safety data led the 2021 Surviving Sepsis Campaign guidelines to suggest initiating vasopressors peripherally to avoid delays, while still recommending CVC placement as soon as feasible for continued vasopressor infusion.^1^

Given these emerging safety data and increasing interest in earlier vasopressor initiation, peripheral vasopressor use has become more prevalent.^9,10,11^ However, most evidence for peripheral vasopressor administration comes from small, single-center studies focused on safety.^5,6,7,8,12^ How clinicians use peripheral vasopressors in practice and their association with patient outcomes remain less clear, particularly given the paucity of multicenter studies and the wide variability in both peripheral vasopressor policies and practices.^9,13^

The multicenter Crystalloid Liberal vs Early Vasopressors in Sepsis (CLOVERS) trial, which compared different sepsis resuscitation strategies and explicitly permitted peripheral vasopressors, presents a unique opportunity to evaluate peripheral vasopressor use in practice.^11^ In this secondary analysis, we evaluate factors influencing vasopressor route selection and the association of peripheral administration with complications and clinical outcomes.

## Methods

This secondary analysis is a prospective, nonrandomized cohort study of peripheral vasopressor administration in CLOVERS (NCT03434028).^11^ CLOVERS was a multicenter, US-based trial comparing an early vasopressor, fluid-restrictive strategy vs a fluid-liberal strategy in patients with sepsis-induced hypotension. Patients from 60 US hospitals were enrolled between March 2018 and February 2022. The trial protocol specified that vasopressors could be administered via central venous access or large PIV at the discretion of the treating team. Each site used its own protocols for peripheral vasopressor administration and extravasation management. Permission for the use of peripheral vasopressors was included in the informed consent process. An evaluation of peripheral vasopressor use was built into the CLOVERS trial design and data collection from the inception of the trial. The study followed the Strengthening the Reporting of Observational Studies in Epidemiology (STROBE) reporting guideline.^14^ This study was reviewed and deemed exempt by the University of Michigan institutional review board.

### Study Population

Patients enrolled in CLOVERS were included in this analysis if they received vasopressors within 24 hours of trial enrollment. Patients were excluded if route of vasopressor administration was unknown or they had central access present prior to enrollment. Central access was defined as a CVC, port-a-cath, or peripherally inserted central catheter. Peripheral vasopressors were defined as any vasopressor administered through a PIV or midline catheter, a specialized long intravascular catheter placed peripherally.^15^

### Data Collection

In the CLOVERS trial, data were collected on time of vasopressor initiation, whether a patient received peripheral vasopressors, and time of central venous access placement between enrollment and study day 3. Consistent with prior studies, vasopressor route was presumed to be central if central access was established and peripheral if the patient was reported as having received peripheral vasopressors and central venous access was not established at the time of vasopressor initiation.^9^

Patient characteristics and vital signs were collected at randomization (baseline). Laboratory results, vasopressor information, and intravenous fluid volumes were collected at baseline and on study days 1 to 3. Vasopressor duration was recorded hourly for the first 24 hours then in days for subsequent use. Peak vasopressor doses were recorded daily on days 1 to 3.

### Exposures

We evaluated route of vasopressor initiation (primary analysis) and route of continuation beyond 6 hours (secondary analysis). We chose a 6-hour time cutoff for continuation based on the Surviving Sepsis Campaign guidelines,^1^ which suggest initiating vasopressors peripherally but transitioning to central access for continued administration.

### Outcomes

We evaluated both complications of peripheral vasopressors and CVC placement and clinical outcomes. Complications were collected to 28 days for all patients who received peripheral vasopressors or had a CVC placed within 3 days of trial enrollment. Collected peripheral vasopressor complications included extravasation and tissue injury. Collected CVC complications included extravasation, catheter-related bloodstream infection, deep vein thrombosis, pneumothorax, blood vessel injury, hemorrhage, hematoma, and arrhythmias. Complications were identified by unblinded study personnel through medical record review and graded using standardized 5-point scales that were specific to each potential complication and based on well-validated surgical complication grading systems.^16^

The primary clinical outcome was 90-day mortality. Secondary outcomes included 72-hour mortality, in-hospital mortality, 28-day intubation, ventilator-free days, new kidney replacement therapy, and intensive care unit (ICU)–free days. We also assessed key process measures: time to vasopressor initiation and fluid volumes received by 6 and 24 hours.

### Statistical Analysis

Data analysis was performed in Stata MP version 18 (Stata Corp) from January 2023 to June 2025. A 2-sided *P* < .05 was considered significant.

#### Main Analysis

We used descriptive statistics to summarize peripheral vasopressor and CVC complications. For peripheral vasopressor infusion, we also calculated the rate of complications per 100 peripheral vasopressor days, defined as total complications divided by total days patients received any peripheral vasopressor therapy. Information about vasopressor administration and route was only collected daily from randomization through study day 3.

We examined trends of peripheral initiation and continuation by study year using the Cochran-Armitage test for trend; 2021 and 2022 were combined given CLOVERS enrollment ended in February 2022.

We compared baseline characteristics across route of vasopressor initiation and continuation using χ^2^ tests for categorical variables and Mann-Whitney *U* tests for continuous variables. Medians (IQRs) were used to summarize continuous variables, which all had skewed distributions based on a skewness and kurtosis test of normality.

We then used mixed multivariable logistic regression models to identify factors associated with peripheral vasopressor initiation and continuation, respectively. To evaluate for variability across study sites, site effects were reported as median odds ratios (mOR), where an mOR of 1.0 means the odds of receiving peripheral vasopressors was similar across sites.^17^ We also report interclass correlation coefficients (ICCs), which represent the proportion of variation explained by site.

For clinical outcomes, we used multivariable models to evaluate the association of (1) route of vasopressor initiation and (2) route of vasopressor continuation with clinical outcomes. We used logistic regression for categorical outcomes, proportional odds models for ventilator and ICU-free days, and linear regression for process outcomes (time to vasopressor initiation, fluid volume). We also performed a survival analysis using a Cox proportional regression model.

All multivariable models included the following covariates that were prespecified based on literature review and clinical experience as important factors that impact selection of vasopressor route or clinical outcomes, and may therefore confound the association of vasopressor route with outcomes: age; sex; Charleson comorbidity index; body mass index; need for respiratory support (invasive ventilation, noninvasive ventilation, or high-flow nasal cannula), Glasgow Coma Score, mean arterial pressure, serum lactate and creatinine values at baseline, location at randomization, and study group (restrictive vs liberal fluid group). The goal of these regression models was to examine the association of vasopressor route with clinical outcomes, adjusting for differences in patients treated with peripheral vs central vasopressors. Missing variables were imputed as outlined in eTable 1 in Supplement 1. Study site was included in all models as a random intercept to account for differences in baseline outcome risk across sites. Sites with less than 10 observations were combined and treated as one site.

#### Sensitivity Analyses

To assess site-level effects, sensitivity analyses were performed using 2 alternative approaches to handling low observation sites: excluding sites with less than 10 observations and combining sites with less than 5 observations. We also performed a mixed multivariable logistic regression model to evaluate the association of race and Sequential Organ Failure Assessment (SOFA) score (eTable 2 in Supplement 1) with peripheral vasopressor initiation. Data on race were collected via medical record review in the CLOVERS trial. Race categories included African American, American Indian and Alaska Native, Asian, Native Hawaiian or Pacific Islander, White, and not reported. American Indian and Alaska Native, Asian, Native Hawaiian or Pacific Islander and not reported races were collapsed into an other race category due to small cell sizes.

To evaluate the association of vasopressor route with 90-day mortality, we performed sensitivity analyses using different site combinations (as previously described), excluding patients with missing variables, adjusting for additional patient variables that were not prespecified (SOFA score, history of heart failure, and history of chronic kidney disease), and using statistical matching techniques.

#### Subgroup Analysis of Patients Who Received Only Peripheral Vasopressors

We also used descriptive statistics to understand baseline characteristics and management practices for patients who received only peripheral vasopressors. This subgroup was defined as patients who were alive but did not have central access placed by 72 hours. Baseline characteristics for these patients were compared with patients who were alive and had central access placed within 72 hours using a χ^2^ test for categorical variables and Mann Whitney* U *test for continuous variables.

## Results

**Figure 1.**
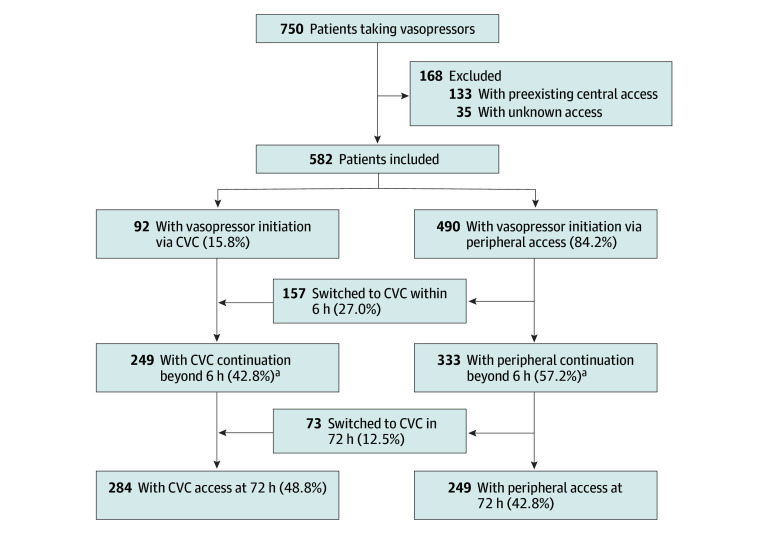
Study Flow Diagram Study flow diagram of patients receiving vasopressors in the Crystalloid Liberal vs Early Vasopressors in Sepsis trial who were included in this study. Route of vasopressor administration is shown with administration through a central venous catheter (CVC; ie, central administration) and administration through a peripheral venous catheter (ie, peripheral administration). Route of vasopressor administration is shown at initiation, continuation beyond 6 hours, and at 72 hours. ^a^Of the 249 individuals with CVC continuation at 6 hours and 333 individuals with peripheral continuation at 6 hours, 49 (8.4%) died within 72 hours.

Of 1563 patients enrolled in CLOVERS, 750 (48.0%) received vasopressors within 24 hours of study enrollment. Of these, 582 (77.6%) met inclusion criteria for this study (Figure 1). Included patients had a median (IQR) age of 63 (52-72) years and a median (IQR) SOFA score of 5 (3-7). Of all patients, 267 (45.9%) were female, 96 (16.5%) were African American, 416 (71.5%) were White, and 70 (12.0%) were another race or had unreported race (Table 1). In total, 490 patients (84.1%) had vasopressors initiated peripherally and 92 (15.8%) had vasopressors initiated centrally. Among the 490 patients initiated on vasopressors peripherally, 230 (46.9%) had central lines placed by day 3, a majority of which (211 patients [91.7%]) were placed on the first day. Overall, 322 patients (55.3%) had central access by day 3.

**Table 1.** Baseline Patient Characteristics by Route of Vasopressor Initiation

Characteristic	Participants, No. (%)			*P* value^a^
	Overall (N = 582)	Peripheral (n = 490)	Central (n = 92)	
Baseline characteristics				
Age, median (IQR) y	63 (53-72)	63 (52-72)	66 (57-72.5)	.17
Sex				
Female	267 (45.9)	222 (45.3)	45 (48.9)	.52
Male	315 (54.1)	268 (54.7)	47 (51.1)	
Race				
African American	96 (16.5)	75 (15.3)	21 (22.8)	.01
White	416 (71.5)	362 (73.9)	54 (58.7)	
Other or not reported^b^	70 (12.0)	53 (10.8)	17 (18.5)	
Admitted from rehabilitation or nursing facility	75 (12.9)	64 (13.1)	11 (12.0)	.77
Body mass index, median (IQR)^c^	26.5 (22.4-31.5)	26.5 (22.4-31.7)	26.3 (22.6-30.4)	.62
Charlson comorbidity index, median (IQR)	4 (2-7)	4 (2-6)	4 (3-7)	.19
Comorbidities				
Hypertension	278 (47.8)	233 (47.6)	45 (48.9)	.89
Diabetes	181 (31.1)	149 (30.4)	32 (34.8)	.65
Malignant neoplasm^d^	133 (22.9)	110 (22.5)	23 (25.0)	.59
Chronic obstructive pulmonary disease	106 (18.2)	93 (19.0)	13 (14.1)	.49
Congestive heart failure	80 (13.8)	67 (13.7)	13 (14.1)	.91
Kidney disease (moderate or severe)	80 (13.8)	61 (12.5)	19 (20.7)	.10
Peripheral vascular disease	56 (9.6)	46 (9.4)	10 (10.9)	.83
Liver disease (moderate or severe)	38 (6.5)	32 (6.5)	6 (6.5)	.97
Study group, fluid-restrictive	365 (62.7)	307 (62.7)	58 (63.0)	.94
Enrolled in emergency department	526 (90.4)	447 (91.2)	79 (85.9)	.23
Baseline vitals and laboratory results^e^				
MAP, median (IQR) mmHg	67 (61-73)	67 (61-73)	65 (58-75)	.62
Heart rate, median (IQR) beats per minute	94 (82-109)	94 (82-108)	98 (83-112)	.20
Respiratory rate, median (IQR) breaths per minute	20 (17-24)	20 (17-24)	20 (18-24)	.37
Glasgow Coma Score, median (IQR)	15 (14-15)	15 (14-15)	15 (14-15)	.21
Lactate, median (IQR) mg/dL	23.4 (14.4-38.7)	23.4 (14.4-37.8)	26.1 (16.2-40.5)	.12
Creatinine, median (IQR) mg/dL	1.6 (1.1-2.6)	1.6 (1.0-2.6)	1.8 (1.2-3.1)	.12
Invasive ventilation	57 (9.8)	46 (9.4)	11 (12.0)	.45
Respiratory support^f^	97 (16.7)	80 (16.3)	17 (18.5)	.26
SOFA score, median (IQR)	5 (3-7)	5 (3-7)	5 (3-8)	.03
Management practices				
Time to vasopressor initiation from hospital arrival,median (IQR) h	4.3 (2.7-7.6)	4.2 (2.6-7.1)	6.3 (3.4-11.3)	<.001
First vasopressor norepinephrine	552 (94.9)	465 (94.9)	87 (94.6)	.51
Peak norepinephrine dose on day 1, median (IQR), µg/kg/min	0.14 (0.06-0.25)	0.12 (0.06-0.24)	0.2 (0.08-0.30)	.007
Peak norepinephrine dose on days 1-3, median (IQR) µg/kg/min	0.14 (0.07,0.3)	0.13 (0.06-0.28)	0.2 (0.1-0.36)	.007
Received a second vasopressor on day 1	114 (19.6)	90 (18.4)	24 (26.1)	.20
Vasopressor beyond 24 h	398 (70.2)	351 (67.6)	77 (83.7)	.002
Total fluids in 24 h, median (IQR) mL^g^	3500 (1218-6579)	3281 (1140-6509)	4048 (2366-6586)	.048
Intensive care unit admission on day 1	510 (87.6)	431 (88.0)	79 (85.9)	.72

Abbreviations: MAP, mean arterial pressure; SOFA, sequential organ failure assessment.

SI conversion factors: To convert creatinine to micromoles per liter, multiply by 88.4; lactate to millimoles per liter, multiply by 0.111.

^a^ *P* values were calculated using χ^2^ tests for categorical variables and Mann-Whitney *U* for continuous variables. A *P* value of .05 was considered significant.

^b^ Included American Indian and Alaska Native, Asian, Native Hawaiian or Pacific Islander, and not reported races.

^c^ Calculated as weight in kilograms divided by height in meters squared.

^d^ Includes solid tumor with or without metastasis, leukemia, and malignant lymphoma.

^e^ Values recorded at the time of randomization.

^f^ Includes mechanical ventilation, high flow nasal oxygen, or noninvasive positive pressure ventilation. Excludes patients on chronic home mechanical ventilation.

^g^ Total fluid from randomization to 24 hours, including crystalloid fluid boluses, albumin, maintenance fluid, blood product, and intravenous medication.

### Complications

Peripheral complications occurred in 3 of 490 patients (0.6%) who received peripheral vasopressors, with an event rate of 0.52 extravasations/100 peripheral vasopressor–days. Two complications were grade 1 (asymptomatic extravasation) and 1 complication was grade 2 (extravasation requiring nonurgent intervention) (Table 2 and eFigure 1 in Supplement 1). There were no ulcerations or skin necrosis. There were 14 complications from CVC placement occurring in 12 of 322 patients (3.7%) who had CVCs placed in the first 3 days of the trial (Table 2).

**Table 2.** Details of Complications From Peripheral Vasopressor Use and Central Venous Catheter Placement Through Day 28^a^

Complication type	Participants by complication grade, No.			
	Overall	Grade 1	Grade 2	Grade 3
Peripheral vasopressor administration complication (n = 490)				
Extravasation	3	2	1	0
CVC placement complication (n = 322)^b^				
Atrial arrhythmia	7	1	4	2
Deep vein thrombosis	3	1	0	2
Ventricular arrhythmia	3	0	1	2
Hematoma	1	1	0	0

Abbreviation: CVC, central venous catheter.

^a^ Complications were collected for any peripheral vasopressor administration and CVC placement that occurred within 72 hours of study enrollment. Complications were tracked out to 28 days and recorded by the study team in the case report forms, using standard grading scales for severity. Grade 1 was defined as asymptomatic and intervention not indicated; grade 2 as nonurgent medical intervention indicated; grade 3 as symptomatic and urgent intervention indicated. There were no documented grade 4 (life-threatening) or grade 5 (death) complications. There were also no documented bloodstream infections, arterial or venous injury, hemorrhage, pneumothorax, or embolism associated with CVC placement in the study population.

^b^ There were 14 CVC placement complications that happened across 12 patients. Two patients had both an atrial and ventricular arrhythmia (both were grade 2 for one patient, while the other patient had a grade 3 ventricular arrhythmia and a grade 2 atrial arrhythmia).

### Peripheral Initiation

**Figure 2.**
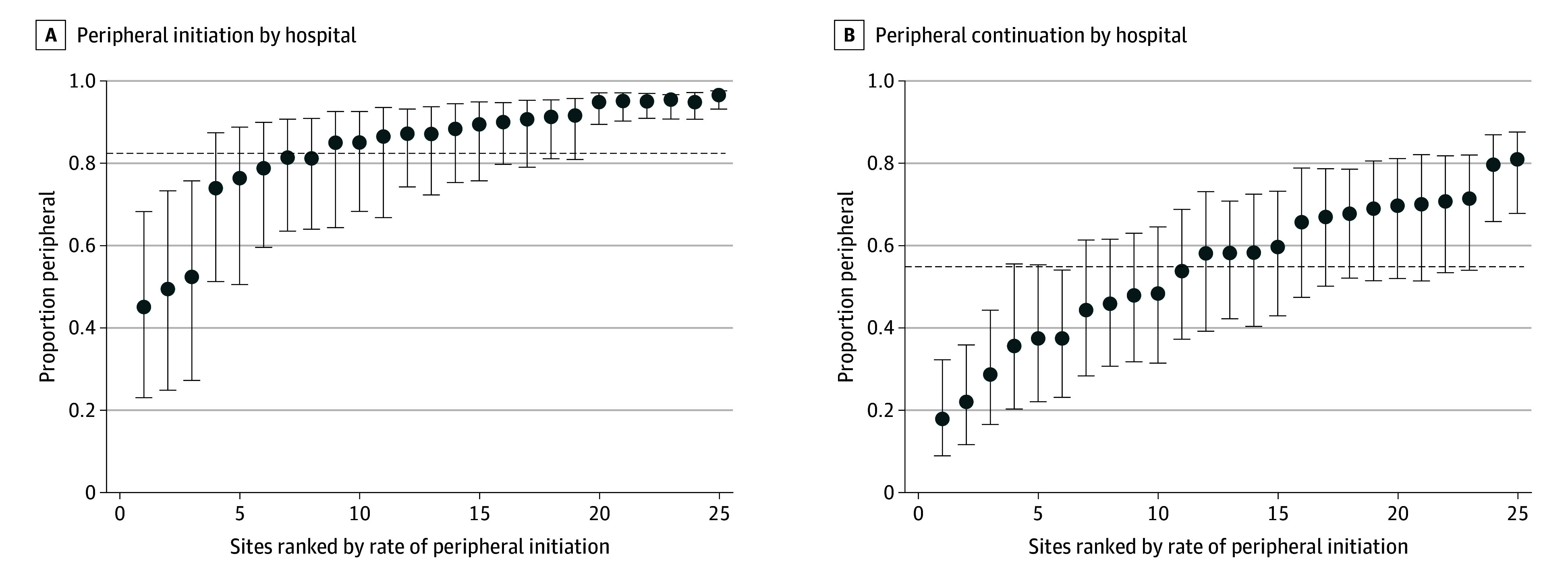
Adjusted Variation in Peripheral Vasopressor Initiation and Continuation Across Sites Caterpillar plots showing variation in adjusted rates of peripheral vasopressor initiation (A) and continuation beyond 6 hours (B) across study sites (hospitals) in the Crystalloid Liberal vs Early Vasopressors in Sepsis trial. Dots reflects the mean rate and the error bars reflect the 95% CI. The population mean is indicated by the dotted line. Sites with less than 10 observations each were combined. Rates were adjusted for following covariables: age, sex, Charleson comorbidity score, body mass index, on noninvasive or invasive mechanical ventilation at baseline, baseline mean arterial pressure, baseline lactate, baseline creatine, randomization location (emergency department vs intensive care unit), and study group.

Rates of peripheral initiation were similar across study groups (fluid-liberal: 183 of 217 participants [84.3%]; fluid-restrictive: 307 of 365 participants [84.1%]; *P* = .94) but decreased over time (eg, 145 of 167 participants [86.8%] in 2018; 54 of 61 participants [73.8%] in 2021-2022; *P* for trend = .01) (eFigure 2 in Supplement 1). Peripheral initiation rates varied across study sites (unadjusted range: 35.7%-100%; adjusted range: 45.7% [95% CI, 23.6%-69.7%] to 98.2% [95% CI, 94.8%-99.3%]) (Figure 2).

In univariable analysis, patients who had vasopressors initiated peripherally were more often White (362 participants [73.9%] vs 54 participants [58.7%]; *P* = .01) and had lower SOFA scores (median [IQR] score, 5 [3-7] vs 5 [3-8]; *P* = .03) (Table 1). Other baseline characteristics, including vitals and laboratory results, were similar between patients with peripheral vs central initiation.

In multivariable analysis, no patient-level factors were independently associated with odds of peripheral initiation (eTable 3 in Supplement 1). While enrollment in the emergency department had a higher point estimate for peripheral initiation, the 95% CI was wide and not significant (adjusted OR [aOR], 1.99; 95% CI, 0.88-4.51). In contrast, study site was associated with greater odds of peripheral initiation (mOR, 3.48; 95% CI, 1.57-5.38), explaining 34% of variation in peripheral vasopressor initiation (ICC = 0.34). Results were similar in sensitivity analyses using different methods to account for low enrollment sites (eTable 4 in Supplement 1) and when including race and SOFA score as covariates (eTable 5 in Supplement 1).

Using peripheral access for vasopressor initiation was associated with shorter time to vasopressor initiation (median [IQR], 4.2 [2.6 to 7.2] vs 6.3 [3.4 to 11.3] hours; adjusted β-coefficient, −2.3 hours; 95% CI, −3.4 to −1.1 hours) and less fluid administration in 24 hours (median [IQR], 3280 [1140-6510] vs 4050 [2370-6590] mL; adjusted β-coefficient, −686 mL; 95% CI, −1278 to −95 mL) (eTable 6 in Supplement 1).

Peripheral vs central vasopressor initiation was not associated with 90-day mortality in the primary analysis (128 participants [26.1%] vs 34 participants [37.0%], aOR 0.67; 95% CI, 0.39-1.16) or in sensitivity analyses (Table 3). Similar results were seen in survival analysis (adjusted hazard ratio, 0.79; 95% CI, 0.54-1.16) (eTable 7 in Supplement 1). After adjustment, peripheral initiation was associated with lower rates of intubation (78 participants [17.8%] vs 32 participants [40.5%]; aOR, 0.31; 95% CI, 0.18-0.56), but statistically comparable mortality at other time points (eg, 72 hours, in-hospital), rates of new kidney replacement therapy, 28-day ventilator intubation, and ICU-free days (eTable 6 in Supplement 1).

**Table 3.** Adjusted 90-Day Mortality by Route of Vasopressor Initiation, Primary and Sensitivity Analyses

Analysis	90-Day Mortality, aOR (95% CI)^a^	Patients, No.	Sites (clusters), No.^b^
Primary analysis^c^	0.67 (0.39-1.16)	582	25
Alternative approaches to combining hospitals			
Drop sites with <10 observations^d^	0.67 (0.36-1.25)	482	24
Combine sites <5 observations^e^	0.67 (0.39-1.18)	582	32
Alternative approaches to multivariable adjustment			
Missing variables dropped^f^	0.63 (0.34-1.18)	429	25
Additional variables added^g^	0.71 (0.41-1.23)	582	25
Matched analyses			
Coarsened exact matching multilevel^h^	0.69 (0.40-1.18)	479	25
Matching on propensity score^i^	0.76 (0.47-1.23)	577	25

Abbreviation: aOR, adjusted odds ratio.

^a^ aOR of mortality based on peripheral vasopressor initiation based on primary analysis and multiple post hoc sensitivity analyses using different approaches combining low-volume hospitals, missing variables, and adjustment.

^b^ Study site was included as a random intercept.

^c^ The primary analysis was a prespecified multivariable mixed logistic regression model adjusting for the following patient factors: age, sex, Charlson Comorbidity Index, body mass index, baseline respiratory support, mean arterial pressure, Glascow Coma Score, lactate, creatinine, study group, and enrollment location, with missing values imputed as described in eTable 1 in Supplement 1. Sites with less than 10 observations each were combined.

^d^ Multivariable mixed logistic regression model adjusting for same covariates as the primary analysis but sites with less than 10 observations each were dropped.

^e^ Multivariable mixed logistic regression model adjusting for same covariates as the primary analysis but sites with less than 5 observations each were combined.

^f^ Multivariable mixed logistic regression model adjusting for same covariates as the primary analysis but missing variables were dropped, rather than imputed. Sites with less than 10 observations were combined as in the primary analysis.

^g^ Multivariable mixed logistic regression model adjusting for the following covariates, with variables that were added and not prespecified: history of congestive heart failure, history of moderate or severe chronic kidney disease, and baseline sequential organ failure assessment score. Variables that were added but prespecified were age, sex, body mass index, Charlson Comorbidity Index, study group, and enrollment location. Sites with less than 10 observations were combined as in the primary analysis.

^h^ Patients were matched using Coarsened Exact Matching on age and sequential organ failure assessment score. Odds of mortality were then calculated using multivariable logistic regression adjusting for the same prespecified covariates as the primary analysis. Site was included as a random intercept; sites with less than 10 observations were combined.

^i^ Patients were matched based on propensity score for odds of peripheral vasopressor initiation, using the same covariates as in the primary analysis. Odds of mortality were then calculated using a multivariable logistic regression model using propensity score and adjusting for the same prespecified covariates as the primary analysis. Site was included as a random intercept; sites with less than 10 observations were combined.

### Peripheral Vasopressor Continuation

Peripheral vasopressors were continued beyond 6 hours in 333 of 582 patients (57.2%) in the study, which represented 68.0% of the 490 patients who had vasopressors initiated peripherally (Figure 1). Rates of peripheral continuation were similar across study groups (fluid-liberal: 119 of 217 participants [54.8%]; fluid-restrictive: 214 of 365 participants [58.6%]; *P* = .37) and over time (*P* for trend = 0.89) (eFigure 2 in Supplement 1). Peripheral continuation rates varied across study sites (unadjusted range: 5.6% to 88.9%; adjusted range: 18.5% [95% CI, 9.5%-33.4%] to 82.5% [95% CI, 69.7%-90.4%]) (Figure 2).

Compared with patients who transitioned to central administration, in univariable analysis, patients with ongoing peripheral vasopressor administration beyond 6 hours were younger (median [IQR] age, 62 [52-71] vs 65 [53-74] years; *P* = .03), more often White (251 of 333 patients [75.4%] vs 175 of 249 patients [66.3%]; *P* = .03), had lower baseline lactate levels (median [IQR], 20.7 [13.5-34.2] vs 27.0 [17.1-41.4] mg/dL [to convert to millimoles per liter multiply by 0.111]; *P* < .001), and were less frequently mechanically ventilated at baseline (23 of 333 patients [6.9%] vs 34 of 249 [13.7%]; *P* = .007) (eTable 8 in Supplement 1). Baseline comorbidities were similar between groups.

In multivariable analysis, the only patient factor independently associated with peripheral continuation was lactate, with peripheral continuation decreasing with higher baseline lactate (aOR, 0.93; 95% CI, 0.87-1.00) (eTable 9 in Supplement 1). Site had a large association with peripheral continuation rates (mOR, 2.35; 95% CI, 1.52-3.19), explaining 19% of variation (ICC = 0.19) (eTable 9 in Supplement 1).

Continuation of vasopressors beyond 6 hours was not associated with 90-day mortality (83 participants [24.9%] vs 49 participants [31.7%]; aOR 0.81; 95% CI, 0.51-1.25) (eTable 10 in Supplement 1). Peripheral continuation was associated lower rates of intubation (44 participants [14.4%] vs 66 participants [31.0%]; aOR, 0.41; 95% CI, 0.24-0.63), less kidney replacement therapy (10 participants [3.0%] vs 20 participants [8.0%]; aOR, 0.38; 95% CI, 0.16-0.88), and more ICU-free days (median [IQR], 26 [24-27] vs 25 [21-26] days; aOR, 0.63; 95% CI, 0.31-0.94). Peripheral continuation was also associated with receiving less total fluid across time points (eTable 6 in Supplement 1).

### Peripheral Vasopressors Only

At 72 hours, 533 of 582 patients (91.6%) were alive. Of these, 284 (53.3%) had a CVC placed ,while 249 (46.7%) had no CVC placed and received only peripheral vasopressors (Figure 1). Patients who received only peripheral vasopressors during the first 72 hours of the trial were less sick at baseline (median [IQR] SOFA score, 4 [2-6] vs 5 [3-7]; *P* = .009), had lower peak day 1 norepinephrine doses (median [IQR] dose, 0.08 [0.05-0.14] vs 0.16 [0.08-0.28] µg/kg/min; *P* < .001), were less likely to receive a second vasopressor (13 patients [5.2%] vs 71 patients [25.0%]; *P* < .001), and were less likely to have vasopressors continued beyond day 1 (135 patients [54.7%] vs 231 patients [81.3%]; *P* < .001) (eTable 11 in Supplement 1).

## Discussion

In this prospective, nonrandomized cohort study of patients enrolled in a multicenter trial of patients with sepsis-induced hypotension, most patients had vasopressors initiated through peripheral access and more than one-half had vasopressors continued peripherally beyond 6 hours. Complication rates associated with peripheral vasopressor administration were low, and vasopressor route was not associated with mortality.

Our results are consistent with other studies showing that peripheral vasopressor administration is common in clinical practice. The CLOVERS trial protocol explicitly permitted peripheral vasopressor initiation to facilitate early vasopressor administration, which may have increased clinician comfort with this approach. However, even outside CLOVERS, peripheral vasopressor initiation is common, reflecting emerging safety data and updated guidelines supporting its use.^1,5,6,7^ For example, a multicenter retrospective study^9^ in Michigan—one of the few multicenter studies of peripheral vasopressors—found that more than two-thirds of patients with sepsis-induced hypotension received peripheral vasopressors. Surprisingly, despite the increasing evidence base supporting the safety of peripheral vasopressors, peripheral initiation in CLOVERS was higher in 2018 compared with the end of the trial, in 2021 to 2022. This decline may reflect changes in trial recruitment patterns over time, especially with the COVID-19 pandemic, rather than broader decline in the use of peripheral vasopressors.

While peripheral vasopressors were widely embraced and included as a consented, study-approved procedure in CLOVERS, rates of peripheral initiation still varied significantly across sites. Similar site-based practice variation was seen in the Michigan study,^9^ suggesting that local institutional culture is an important factor underlying peripheral vasopressor practices. While White patients were more likely to receive peripheral vasopressors in univariable analysis, this finding was not significant after adjusting for study site, suggesting that this difference is due to hospital-level differences in vasopressor practices. The finding of large hospital-level variation highlights the need for standardized protocols to reduce variability in peripheral vasopressor use across institutions.

Future work should address both the route of vasopressor initiation and safety of continued peripheral infusion. While CLOVERS permitted peripheral initiation to facilitate early administration of vasopressors, the trial protocol did not comment on continued peripheral infusion. Nevertheless, more than one-half of the patients in this study had vasopressors continued peripherally beyond 6 hours, a rate that remained stable over time even as the rate of peripheral initiation declined. Consistent with prior studies, we also found that a large proportion of patients—more than 40%—did not receive central access by day 3.^8,9,12,18^ As expected, patients who received only peripheral vasopressors tended to be less sick at baseline and were often taking a single vasopressor, with only one-half continuing vasopressors beyond the first day. However, the optimal thresholds for transitioning from peripheral to central administration remain uncertain because current studies and institutional policies vary widely in their recommendations.^5,6,7,8,13^ Future studies should address these gaps to provide more consistent and evidence-based recommendations.

Importantly, even with the widespread use of peripheral vasopressors in CLOVERS, adverse events were rare (0.6%) and low-grade. This adverse event rate is notably lower than the 3% to 7% extravasation rates reported in prior safety studies.^5,6,7^ Complications in CLOVERS were collected through medical record review rather than direct observation. While this approach may underestimate minor extravasations, it provides reassurance that major complications are uncommon. In contrast, the 3.7% CVC complication rate in CLOVERS is similar to other studies, confirming that CVC placement carries inherent risks.^19,20^

While most studies of peripheral vasopressors have focused on safety,^8,12^ it is also important to understand the impact of this common practice on patient outcomes, including mortality. While it is unlikely that peripheral vasopressors directly cause mortality, the use of peripheral vasopressors could have other unmeasured effects on care delivery. Such effects could theoretically increase mortality (eg, by enabling reduced monitoring or impairing recognition of progressive shock) or decrease mortality (eg, by shortening shock duration, as in The Comparison Between Early Norepinephrine Use and Standard Treatment During Severe Sepsis and Septic Shock Resuscitation trial, where early administration of norepinephrine, mostly peripherally, was associated with improved shock control).^21^ To assess these potential unmeasurable effects of peripheral vasopressor use, we evaluated the association of vasopressor route with 90-day mortality. Consistent with the multicenter Michigan study,^9^ we found no association of peripheral vasopressor initiation with mortality. Importantly, these results were similar across multiple sensitivity analyses aimed at minimizing residual confounding, including statistical matching techniques, suggesting there is no association of early vasopressor route with mortality and providing reassurance that the broader practices associated with peripheral vasopressor use are unlikely to be harmful. While several secondary clinical outcomes were less common in the peripheral vasopressor group, these findings likely reflect differences between patients receiving peripheral vs central vasopressors. This is particularly true for route of vasopressor continuation. For example, the association of peripheral continuation with lower rates of intubation and kidney replacement therapy likely reflects that patients who are intubated or on dialysis often require central venous access rather than representing a true association of vasopressor route with outcomes. These results build on existing safety data and suggest that early peripheral vasopressors are at least unlikely to increase mortality and may offer practical advantages in the management of sepsis-induced hypotension, such as faster vasopressor initiation.

### Limitations

This study has several limitations. First, as a nonrandomized cohort study, it is at risk for residual confounding, despite our efforts to address this risk by using evidence-based, prespecified covariates and multiple modeling approaches. Second, the CLOVERS trial encouraged peripheral vasopressor initiation. While this limits generalizability, it provides insight into clinical practices that might emerge under guidelines promoting peripheral vasopressor use. Third, in CLOVERS data collection, no differentiation was made between PIV vs midline as the route of peripheral vasopressor administration, which may influence the interpretation of safety outcomes. Fourth, while the identification and grading of complications were standardized, complications were assessed by unblinded study personnel which may have introduced detection bias. Furthermore, complications were collected through retrospective medical record review where it can be difficult to determine whether certain complications, such as arrhythmias, were the result of CVC placement or the effect of critical illness or vasopressor therapy more generally, which could have biased estimates of CVC complication rates.

## Conclusions

In this prospective cohort study of patients enrolled in the CLOVERS trial, early peripheral vasopressor use was common with low complication rates and no significant association with mortality. These findings support the safety and feasibility of early peripheral vasopressors in the management of sepsis-induced hypotension. However, substantial variation in practice across study sites highlights the influence of institutional culture on vasopressor administration, underscoring the need for work to standardize both the initiation and continuation of peripheral vasopressors. Future studies should address key unanswered questions, including the safety of prolonged peripheral vasopressor use, dose thresholds for transitioning to central access, and the impact of catheter types on safety and efficacy. Addressing these gaps in knowledge will help define clinical guidelines and enhance consistency in sepsis resuscitation practices.

## Notes

Supplement 1. **eTable 1.** Approach to missingness **eTable 2.** Sequential organ failure assessment (SOFA) score calculation catheter placement **eFigure 1.** 28-day complications from peripheral vasopressor administration and central **eFigure 2.** Trends in peripheral vasopressor initiation and continuation over time **eTable 3.** Factors associated with peripheral vasopressor initiation **eTable 4.** Sensitivity analysis populations and study site effect **eTable 5.** Sensitivity analysis of factors associated with peripheral vasopressor initiation including race and SOFA score **eTable 6.** Multivariable mixed-effects regression models of secondary and process outcomes (vasopressor and fluid practices) by route of vasopressor initiation and continuation **eTable 7.** Survival analysis by vasopressor route **eTable 8.** Baseline patient characteristics, by route of vasopressor continuation beyond 6 hours **eTable 9.** Factors associated with peripheral vasopressor continuation beyond 6 hours **eTable 10.** Adjusted 90-day mortality by route of vasopressor continuation, primary and sensitivity analyses **eTable 11.** Characteristics of patients who received only peripheral vasopressors

Supplement 2.
Nonauthor Collaborators

Supplement 3.
Data Sharing Statement
