# Supplementary material for: Peripheral Vasopressor Use in Early Sepsis-Induced Hypotension
Source: JAMA Netw Open. 2025 Aug 27;8(8):e2529148. doi: 10.1001/jamanetworkopen.2025.29148 (PMC12391982; doi:10.1001/jamanetworkopen.2025.29148)
Supplement: Supplement 1. — eTable 1. Approach to missingness eTable 2. Sequential organ failure assessment (SOFA) score calculation catheter placement eFigure 1. 28-day complications from peripheral vasopressor administration and central eFigure 2. Trends in peripheral vasopressor initiation and continuation over time eTable 3. Factors associated with peripheral vasopressor initiation eTable 4. Sensitivity analysis populations and study site effect eTable 5. Sensitivity analysis of factors associated with peripheral vasopressor initiation including race and SOFA score eTable 6. Multivariable mixed-effects regression models of secondary and process outcomes (vasopressor and fluid practices) by route of vasopressor initiation and continuation eTable 7. Survival analysis by vasopressor route eTable 8. Baseline patient characteristics, by route of vasopressor continuation beyond 6 hours eTable 9. Factors associated with peripheral vasopressor continuation beyond 6 hours eTable 10. Adjusted 90-day mortality by route of vasopressor continuation, primary and sensitivity analyses eTable 11. Characteristics of patients who received only peripheral vasopressors [file jamanetwopen-e2529148-s001.pdf]

Supplemental Online Content

Munroe ES, Co IN, Douglas I, et al; for the NHLBI Petal Network. Peripheral vasopressor use in early sepsis-induced hypotension. *JAMA Netw. Open*. 2025;8(8): e2529148. doi:10.1001/jamanetworkopen.2025.29148

eTable 1. Approach to missingness

eTable 2. Sequential Organ Failure Assessment (SOFA) score calculation

catheter placement

eFigure 1. 28-day complications from peripheral vasopressor administration and central

eFigure 2. Trends in peripheral vasopressor initiation and continuation over time

eTable 3. Factors associated with peripheral vasopressor initiation

eTable 4. Sensitivity analysis populations and study site effect

eTable 5. Sensitivity analysis of factors associated with peripheral vasopressor initiation including race and SOFA score

eTable 6. Multivariable mixed-effects regression models of secondary and process outcomes (vasopressor and fluid practices) by route of vasopressor initiation and continuation

eTable 7. Survival analysis by vasopressor route

eTable 8. Baseline patient characteristics, by route of vasopressor continuation beyond 6 hours

eTable 9. Factors associated with peripheral vasopressor continuation beyond 6 hours

eTable 10. Adjusted 90-day mortality by route of vasopressor continuation, primary and sensitivity analyses

eTable 11. Characteristics of patients who received only peripheral vasopressors

This supplemental material has been provided by the authors to give readers additional information about their work.

© 2025 Munroe ES et al. *JAMA Network Open*.

**eTable 1.** Approach to Missing Variables		
**Variable**	**Number missing (%)**	**Imputed value**
Creatinine, baseline	8 (1.4%)	Normal (1.0 mg/dL)
Lactate, baseline	58 (10.0%)	Normal (1.5 mmol/L)
Bilirubin, baseline	94 (16.2%) missing at baseline;<br>60 (10.3%) missing at baseline and day 1	If missing at baseline, day 1 value was used. If no day 1 value, imputed as normal (1.0 mg/dL)
Platelets, baseline	2 (0.3%) missing at baseline, 0 (0%) missing day 1	If missing at baseline, day 1 value was used.
Mean Arterial Pressure	3 (0.5%)	Population mean (67.7 mmHg)
PaO2 or SpO2:FiO2	23 (4.0%)	Imputed as 400
Charleston Comorbidity index	1 (0.2%)	0
Body Mass Index	18 (3.1%)	Mean BMI by sex (26.7 for males, 29.0 for females)
Glascow Coma Score	87 (14.9%)	Normal (15)

**eTable 2.** SOFA score calculation					
**Points**	**Cardiovascular** (mean arterial pressure; MAP)	**Respiratory** (P:F or S:F+)	**Hematologic** (Platelets x $10^3/\mu L$)	**Liver** (Bilirubin, mg/dL)	**Renal** (Creatinine, mg/dL)
**0**	MAP ≥ 70 mmHg	≥400	≥150	<1.2	<1.2
**+1**	MAP < 70 mmHg	<400	<150	1.2-1.9	1.2-1.9
**+2**	**	<300	<100	2.0-5.9	2.0-3.4
**+3**	On any vasopressor**	<200 and on mechanical ventilation	<50	6.0-11.9	3.5-4.9
**+4**	**	<100 and on mechanical ventilation	<20	≥12	≥5

For this analysis, the Sequential Organ Function Assessment (SOFA) score was calculated using baseline values. Missing values were imputed, as described in eTable 1.

**The SOFA score uses vasopressor dose to provide points to patients on vasopressors with 2 points assigned for dopamine < 5 or dobutamine, 3 points assigned for dopamine >5, epinephrine or norepinephrine > 0.1, and 4 points assigned for dopamine >15 or norepinephrine or epinephrine > 0.1 (with units in mcg/kg/min). However, exact vasopressor doses at baseline were not available. No patients were on dobutamine and only 2 patients were on dopamine. Therefore, all patients on vasopressors at baseline were assigned a score of +3 for the Cardiovascular system.

+PaO2:FiO2 was used when available. When not available, SpO2:FiO2 was used instead.

© 2025 Munroe ES et al. *JAMA Network Open*.

eFigure 1. 28-day complications from peripheral vasopressor administration and central catheter placement.

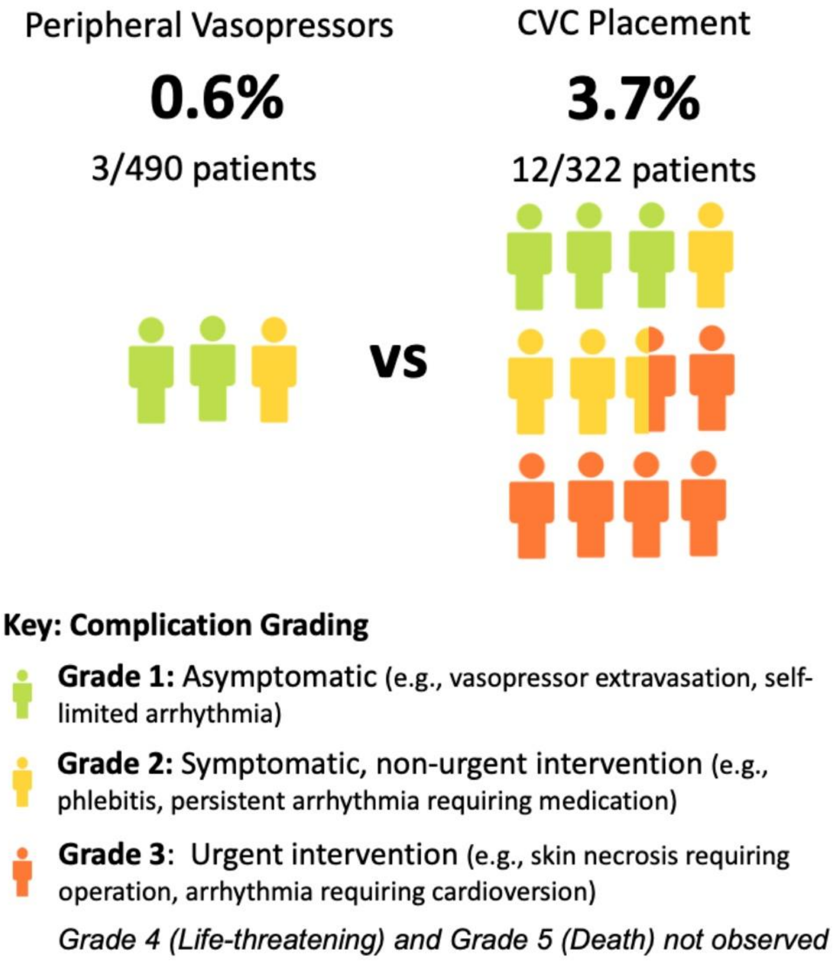

© 2025 Munroe ES et al. *JAMA Network Open*.

eFigure 2. Trends in peripheral vasopressor initiation and continuation over time

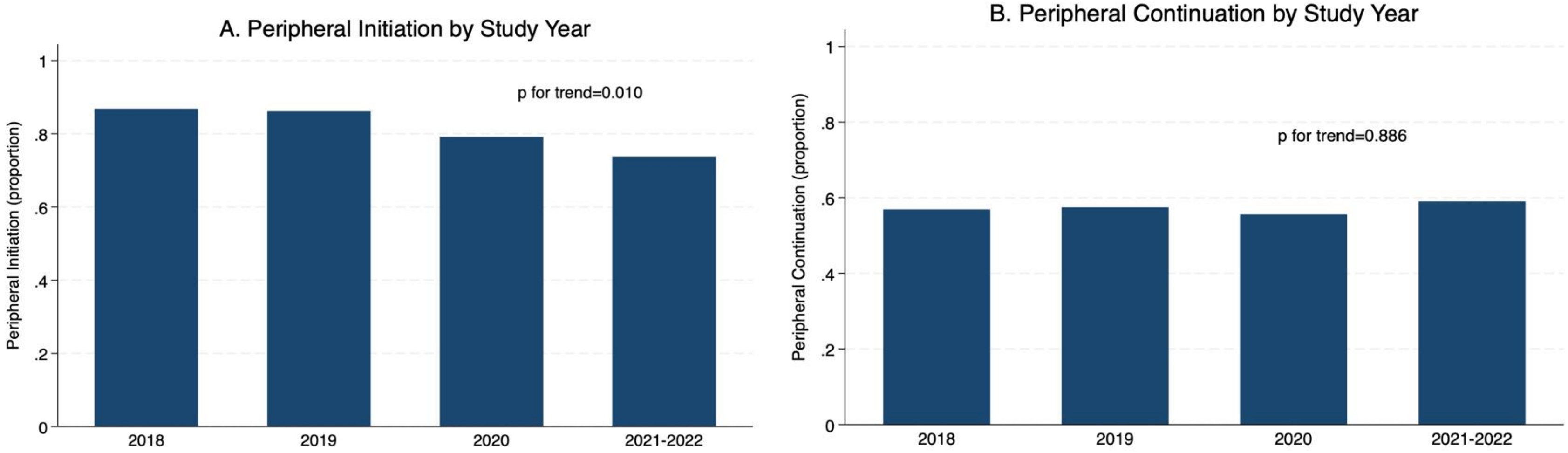

eFigure 2 Legend. Trends in proportion of patients receiving vasopressors who had vasopressors initiated peripherally (A) and continued peripherally beyond 6 hours (B) over the CLOVERS study period. Given the small sample size in 2022 when the study ran for 1 month (N=6), years 2021 and 2022 were combined. P-value for trend was calculated using the Cochran-Armitage test.

© 2025 Munroe ES et al. *JAMA Network Open*.

**eTable 3:** Factors associated with peripheral vasopressor initiation			
**Variable**	**aOR of peripheral initiation**	**95% CI**	**p-value**
Age, per year	1.00	0.98, 1.03	0.669
Sex, female	0.85	0.51, 1.43	0.548
Charleson comorbidity index	0.97	0.87, 1.07	0.492
BMI, per kg/m^2	1.01	0.98, 1.04	0.629
On respiratory support* at baseline	0.99	0.44, 2.24	0.977
Baseline MAP, mmHg	1.01	0.99, 1.03	0.412
Baseline GCS	0.99	0.89, 1.10	0.811
Baseline Lactate, mmol/L	0.99	0.91, 1.07	0.766
Baseline Creatinine, mg/dL	0.93	0.80, 1.07	0.323
Fluid-Restrictive Study Arm	0.92	0.54, 1.56	0.760
Enrolled in ED	1.99	0.88, 4.51	0.101
Hospital effect	mOR: 3.47	1.57, 5.38	<0.001

eTable 3 Legend: Adjusted odds of peripheral vasopressor initiation was determined using a multivariable logistic regression model including all patient factors listed in the first column as fixed effects and hospital as a random intercept. Baseline values refer to values at the time of randomization. Hospitals with <10 observations each were combined for primary analysis. N=582 patients.

Definitions: aOR= adjusted odds ratio, CI= confidence interval, BMI= body mass index, MAP= mean arterial pressure, GCS= glascow coma score, ED= Emergency department, ICC= interclass correlation coefficient, mOR= median ddds ratio

*Includes mechanical ventilation, high flow nasal oxygen or non-invasive positive pressure ventilation. Excludes patients on chronic home mechanical ventilation.

© 2025 Munroe ES et al. *JAMA Network Open*.

**eTable 4.** Sensitivity analysis populations and site effect				
	**Peripheral Initiation**		**Peripheral Continuation**	
	**ICC**	**Median OR**	**ICC**	**Median OR**
**Primary analysis** (Combine sites with <10 observations)	0.34	3.47 (1.57, 5.38)	0.19	2.35 (1.52, 3.19)
Drop sites with <10 observations	0.36	3.69 (1.54, 5.83)	0.21	2.43 (1.53, 3.34)
Combine sites < 5 observations	0.33	3.33 (1.70, 4.96)	0.18	2.28 (1.56, 3.00)

eTable 4 Legend: Site effect on route of vasopressor initiation and continuation within the primary analysis population and sensitivity analysis populations, using different approaches to combining low volume sites. In the primary analysis, sites with <10 observations each were combined. The following sensitivity analysis populations were evaluated: 1) hospitals with <10 observations each were dropped (N=482) and 2) hospitals with < 5 observations each were combined (N=582).

Site effects are reported as median odds ratio (mOR), where a mOR of 1.0 means the odds of receiving peripheral vasopressors was similar across sites. The larger the mOR, the more site-level variation explains variation in vasopressor route. We also report interclass correlation coefficient (ICC), which represents the amount of variation explained by hospital.

© 2025 Munroe ES et al. *JAMA Network Open*.

**eTable 5.** Sensitivity analysis of factors associated with peripheral vasopressor initiation			
**Variable**	**aOR of peripheral initiation**	**95% CI**	**p-value**
Age, per year	1.00	0.98, 1.03	0.420
Race			
White	Ref	Ref.	Ref.
African American	0.80	0.39, 1.64	0.535
Other	0.85	0.38, 1.89	0.683
Sex, female	0.86	0.51, 1.43	0.588
Charleson comorbidity index	0.96	0.86, 1.06	0.384
BMI, per kg/m^2	1.01	0.98, 1.04	0.602
SOFA score	0.91	0.81, 1.02	0.093
On respiratory support* at baseline	1.06	0.45, 2.50	0.899
Baseline MAP, mmHg	1.01	0.99, 1.04	0.287
Baseline GCS	0.95	0.84, 1.07	0.375
Baseline Lactate, mmol/L	0.99	0.91, 1.08	0.872
Baseline Creatinine, mg/dL	1.01	0.85, 1.19	0.940
Fluid-Restrictive Study Arm	0.90	0.53, 1.54	0.706
Enrolled in ED	1.76	0.75, 4.15	0.194
Hospital effect	mOR: 3.28	1.65, 4.91	<0.001

eTable 5 Legend: Adjusted odds of peripheral vasopressor initiation including pre-specified variables used in the primary analysis and two additional variables in italics (race, SOFA score). The adjusted odds ratio was calculated using multivariable mixed logistic regression model including all patient factors listed in the first column as fixed effects and hospital as a random intercept. Baseline values refer to values at the time of randomization. Hospitals with <10 observations each were combined for primary analysis. N=582 patients.

Definitions: aOR= adjusted odds ratio, CI= confidence interval, BMI= body mass index, MAP= mean arterial pressure, GCS= glascow coma score, ED= Emergency department, ICC= interclass correlation coefficient, mOR= median ddds ratio

*Includes mechanical ventilation, high flow nasal oxygen or non-invasive positive pressure ventilation. Excludes patients on chronic home mechanical ventilation.

**eTable 6:** Multivariable mixed-effects regression models of secondary and process outcomes (vasopressor and fluid practices) by route of vasopressor initiation and continuation						
	**Initiation**			**Continuation**		
	**Peripheral N=490**	**Central N=92**	**Adjusted OR (95% CI)**	**Peripheral N=333**	**Central N=249**	**Adjusted OR (95% CI)**
**Primary outcome:** 90-day mortality, N(%)	128 (26.1%)	34 (37.0%)	0.67 (0.39, 1.16)	83 (24.9%)	79 (31.7%)	0.80 (0.51, 1.25)
**Secondary outcomes**						
Early (72-hour) mortality, N(%)	38 (7.8%)	11 (12.0%)	0.68 (0.30, 1.52)	23 (6.9%)	26 (10.4%)	0.90 (0.45, 1.79)
In-hospital mortality, N(%)	83 (16.9%)	24 (26.1%)	0.63 (0.34, 1.17)	51 (15.3%)	56 (22.5%)	0.74 (0.44, 1.23)
Intubated between randomization and day 28, N(%)	78 (17.8%)	32 (40.5%)	0.31 (0.18, 0.56)	44 (14.4%)	66(31.0%)	0.40 (0.24, 0.63)
Ventilator-free days to day 28, median (IQR)	28 (22, 28)	26 (0, 28)	0.59 (0.13, 1.05)	28 (26, 28)	27 (0, 28)	0.51 (0.14, 0.88)
New renal replacement therapy within 28 days of randomization, N(%)	22 (4.5%)	8 (8.7%)	0.55 (0.21, 1.43)	10 (3.0%)	20 (8.0%)	0.38 (0.16, 0.88)
ICU-free days, median (IQR)	25 (23, 27)	24 (20, 26)	0.72 (0.30, 1.12)	26 (24, 27)	25 (21, 26)	0.63 (0.31, 0.94)
**Process Outcomes**			Beta coefficient (95%CI)			Beta coefficient (95% CI)
Time to vasopressor initiation (hrs)	4.2 (2.6, 7.2)	6.3 (3.4, 11.3)	-2.3 (-3.4, -1.1)	4.2 (2.5, 7.1)	5.0 (3.0, 8.4)	-0.6 (-1.5, 0.3)
Total fluid+ in 6 hrs* (mL)	1,030 (340, 2,550)	1,230 (500, 2,230)	20 (-188, 228)	1000 (270, 2380)	1280 (470, 2840)	-206 (-360, -52)
Total fluid+ in 24 hrs* (mL)	3280 (1140, 6510)	4050 (2370, 6590)	-686 (-1278, -95)	2970 (870, 6080)	4380 (1900, 7110)	-759 (-1197, -320)

eTable 6 Legend: Patient outcomes by route of vasopressor initiation and continuation. Raw outcomes are presented as N(%) or median (IQR).
*Adjusted odds ratios (aOR) were determined using multivariable logistic regression (for dichotomous outcomes), proportional odds models (for ventilator-free and ICU-free days), and linear regression (for time to vasopressor initiation and total fluid volumes). Estimation was performed using maximum likelihood for logistic regression and proportional odds models and restricted maximum likelihood for linear regression models. The following covariables included as fixed effects: age, sex, Charleson co-morbidity score, body mass index, on non-invasive or invasive mechanical ventilation at baseline, baseline mean arterial pressure, baseline lactate, baseline creatine, randomization location (ED vs ICU), and study arm. In all models, enrollment site was included as a random intercept and sites with <10 observations each were combined.
+Total fluid includes crystalloid fluid boluses, albumin, maintenance fluid, blood product, and IV medication measured in milliliters
*Total fluid volumes are measured from randomization to hour 6 and hour 24, respectively.

© 2025 Munroe ES et al. *JAMA Network Open*.

**eTable 7.** Survival analysis by vasopressor route		
	**Unadjusted hazard ratio (95% CI)**	**Adjusted hazard ratio* (95% CI)**
Peripheral initiation	0.65 (0.45, 0.95)	0.79 (0.54, 1.16)
Peripheral continuation	0.75 (0.55, 1.02)	0.93 (0.67, 1.31)
eTable 7. Cox proportional regression model for 90-day mortality by route of vasopressor initiation and continuation.<br>*Adjusted for pre-specified co-variates used in primary mortality models		

© 2025 Munroe ES et al. *JAMA Network Open*.

**eTable 8**. Baseline patient characteristics, by route of vasopressor continuation beyond 6 hours				
	Overall<br>N=582	Peripheral<br>N= 333	Central<br>N= 249	P-value
**Baseline characteristics**				
Age, years, median (IQR)	63 (53-72)	62 (52, 71)	65 (53, 74)	0.026
Sex, female, N(%)	267 (45.9%)	152 (45.7%)	115 (46.2%)	0.897
Race, N(%)				
White	416 (71.5%)	251 (75.4%)	165 (66.3%)	0.029
African-American	96 (16.5%)	51 (15.3%)	45 (18.1%)	
Other/Not reported	70 (12.0%)	31 (9.3%)	39 (15.7%)	
Admitted from rehab or nursing facility, N(%)	75 (12.9%)	41 (12.3%)	34 (13.7%)	0.633
BMI, kg/m^2, median (IQR)	26.5 (22.4, 31.5)	26.5 (22.7, 32.5)	26.5 (22.3, 30.2)	0.122
Charlson comorbidity index, median (IQR)	4 (2, 7)	4 (2,7)	4 (2, 7)	0.755
Co-morbidities, N(%)				
Hypertension,	278 (47.8%)	146 (43.8%)	132 (53.0%)	0.067
Diabetes	181 (31.1%)	107 (32.1%)	74 (29.7%)	0.558
Malignancy[⊥]	133 (22.9%)	84 (25.2%)	49 (19.7%)	0.115
COPD	106 (18.2%)	66 (19.8%)	40 (16.1%)	0.343
Congestive heart failure	80 (13.8%)	45 (13.5%)	35 (14.1%)	0.677
Kidney disease (moderate/severe)	80 (13.8%)	40 (12.0%)	40 (16.1%)	0.261
Peripheral vascular disease	56 (9.6%)	30 (9.0%)	26 (10.4%)	0.585
Liver disease (moderate/severe)	38 (6.5%)	24 (7.2%)	14 (5.6%)	0.643
Study arm, fluid-restrictive, N(%)	365 (62.7%)	214 (64.3%)	151 (60.6%)	0.371
Enrolled in ED, N(%)	526 (90.4%)	306 (91.9%)	220 (88.4%)	0.301
**Baseline vitals and labs***				
MAP, mmHg, median (IQR)	67 (61, 73)	67 (61, 74)	65 (60, 73)	0.100
Heart rate, beats per minute, median (IQR)	94 (82, 109)	92 (80,107)	98 (83, 110)	0.033
Respiratory rate, breaths per minute, median (IQR)	20 (17, 24)	19 (16, 23)	20 (18, 25)	0.027
Glascow Coma Score, median (IQR)	15 (14, 15)	15 (14, 15)	15 (14, 15)	0.141
Lactate, mmol/L, median (IQR)	2.6 (1.6, 4.3)	2.3 (1.5, 3.8)	3 (1.9. 4.6)	<0.001
Creatinine, mg/dL, median (IQR)	1.6 (1.1, 2.6)	1.6 (1.1, 2.4)	1.6 (1.0, 2.9)	0.356
On invasive ventilation, N(%)	57 (9.8%)	23 (6.9%)	34 (13.7%)	0.007
On respiratory support**, N(%)	97 (16.7%)	47 (14.1%)	50 (20.1%)	0.056
SOFA score, median (IQR)	5 (3,7)	4 (3, 7)	5 (3, 7)	0.155
**Vasopressor characteristics**				
Time to vasopressor initiation from hospital arrival in hours, median (IQR)	4.3 (2.7, 7.6)	4.2 (2.5, 7.1)	5.0 (3.0, 8.4)	0.038
First vasopressor norepinephrine, N(%)	552 (94.9%)	318 (95.5%)	234 (93.4%)	0.404
Peak norepinephrine dose (mcg/kg/min), day 1, median (IQR)	0.14 (0.06, 0.25)	0.11 (0.05, 0.2)	0.18 (0.08, 0.33)	0.195
Received a second vasopressor on day 1, N(%)	114 (19.6%)	38 (11.4%)	76 (30.5%)	<0.001
On vasopressor beyond 24 hours, N(%)	398 (70.2%)	203 (62.7%)	195 (80.3%)	<0.001
Total fluids in 24 hours, mL[+], median (IQR)	3500 (1218, 6579)	2966 (869, 6077)	4378 (1896, 7112)	<0.001
ICU admission on day 1, N(%)	510 (87.6%)	296 (88.9%)	214 (85.9%)	0.485

© 2025 Munroe ES et al. *JAMA Network Open*.

eTable 7 Legend: Baseline patient characteristics by route of vasopressor continuation (beyond 6 hours), peripheral vs central. Data are presented as median (IQR) or N (%). P-values were calculated using chi-squared test for categorical variables and Mann Whitney U test for continuous variables. A p-value of 0.05 was considered significant.
⊥ Malignancy includes solid tumor with or without metastasis, leukemia, and malignant lymphoma.
*Baseline vital signs and labs were the values recorded at the time of randomization
**Respiratory support includes mechanical ventilation, high flow nasal oxygen or non-invasive positive pressure ventilation. Excludes patients on chronic home mechanical ventilation.
+Total fluid from randomization to 24 hours, including crystalloid fluid boluses, albumin, maintenance fluid, blood product, and IV medication.
Definitions: IQR= interquartile range, BMI= body mass index, COPD= chronic obstructive pulmonary disease, ED= emergency department, MAP= mean arterial pressure, ICU= intensive care unit, SOFA= sequential organ failure assessment

© 2025 Munroe ES et al. *JAMA Network Open*.

**eTable 9:** Factors associated with peripheral vasopressor continuation beyond 6 hours		
**Variable**	**aOR of peripheral continuation (95% CI)**	**p-value**
Age, per year	0.99 (0.97, 1.00)	0.100
Sex, female	0.98 (0.67, 1.45)	0.938
Charleson comorbidity index	1.08 (1.00, 1.16)	0.056
BMI, per kg/m^2	1.02 (1.00, 1.04)	0.122
On respiratory support at baseline*	0.77 (0.42, 1.41)	0.398
Baseline MAP, mmHg	1.01 (0.99, 1.03)	0.305
Baseline GCS	1.01 (0.93, 1.09)	0.878
Baseline Lactate, mmol/L	0.93 (0.87, 1.00)	0.048
Baseline Creatinine, mg/dL	0.92 (0.83, 1.02)	0.120
Fluid-Restrictive Study Arm	1.16 (0.79, 1.70)	0.456
Enrolled in ED	1.46 (0.76, 2.82)	0.257
Hospital effect (median OR)	2.35 (1.52, 3.19)	<0.001

eTable 8 Legend. Adjusted odds of peripheral vasopressor continuation beyond 6 hours was determined using a multivariable logistic regression model including all patient factors listed in the first column as fixed effects and hospital as a random intercept. Baseline values refer to values at the time of randomization. Hospitals with <10 observations each were combined for primary analysis. N=582 patients.

Definitions: aOR= adjusted odds ratio, CI= confidence interval, BMI= body mass index, MAP= mean arterial pressure, GCS= glascow coma score, ED= Emergency department, ICC= interclass correlation coefficient, mOR= median ddds ratio

* Includes mechanical ventilation, high flow nasal oxygen or non-invasive positive pressure ventilation. Excludes patients on chronic home mechanical ventilation.

© 2025 Munroe ES et al. *JAMA Network Open*.

**eTable 10.** Adjusted 90-day mortality by route of vasopressor continuation, primary and sensitivity analyses			
	**aOR 90-day mortality (95% CI)**	**Number of patients**	**Number of hospitals (clusters)**
**Primary analysis**			
A. Primary analysis	0.80 (0.51, 1.25)	582	25
**Alternative approaches to combining hospitals**			
B. Drop sites with <10 observations	0.85 (0.52, 1.38)	482	24
C. Combine sites < 5 observations	0.82 (0.52, 1.29)	582	32
**Alternative approaches to multivariable adjustment**			
D. Missing variables dropped	0.71 (0.42, 1.19)	429	25
E. Post-hoc analysis	0.69 (0.44, 1.08)	582	25
**Matched analyses**			
G. CEM Matching	0.70 (0.49, 0.99)	542	25
I. Matching on propensity score	0.67 (0.45, 0.99)	582	25

eTable 9 Legend. Adjusted odd ratio of mortality based on peripheral vasopressor initiation based on primary analysis and multiple post-hoc sensitivity analyses using different approaches to combining low-volume hospitals, missing variables, and adjustment.

A. The primary analysis was a prespecified multivariable mixed logistic regression models adjusting for the following patient factors: age, sex, Charlson Comorbidity Index, BMI, baseline respiratory support, mean arterial pressure (MAP), glascow coma score, lactate, creatinine, study arm, and enrollment location, with missing values imputed as described in eTable 1. Study site was included as a random intercept. Sites with <10 observations each were combined.
B. Multivariable mixed logistic regression model adjusting for same covariates as (A) but sites with <10 observations each were dropped.
C. Multivariable mixed logistic regression model adjusting for same covariates as (A) but sites with < 5 observations each were combined
D. Multivariable mixed logistic regression model adjusting for same covariates as (A) but missing variables were dropped, rather than imputed. Sites with <10 observations were combined as in the primary analysis.
E. Multivariable mixed logistic regression model adjusting for the following co-variates, with those added post-hoc in italics: age, sex, BMI, Charlson Comorbidity Index, *history of congestive heart failure, history of moderate/severe chronic kidney disease*, *baseline SOFA score*, study arm, and enrollment location. Sites with <10 observations were combined as in the primary analysis
F. Patients were matched using Coarsened Exact Matching (CEM) on age and SOFA score. Odds of mortality were then calculated using multivariable logistic regression adjusting for the same pre-specified covariates the primary analysis (A). Site was included as a random intercept; sites with <10 observations were combined
G. Patients were matched based on their propensity score for odds of peripheral vasopressor initiation, using the same covariates as in the primary analysis (A). Odds of mortality were then calculated using a multivariable logistic regression model using propensity score and adjusting for the same pre-specified covariates the primary analysis (A). Site was included as a random intercept; sites with <10 observations were combined.

© 2025 Munroe ES et al. *JAMA Network Open*.

eTable 11. Characteristics of patients who received only peripheral vasopressors			
	Patients alive and without central access by 72 hours (Peripheral vasopressors only), N=249	Patients alive who received central access by 72 hours, N=284	p-value
**Patient characteristics**			
Age, years, median (IQR)	62 (52, 70)	64 (52, 73)	0.049
Sex, female, N(%)	106 (42.6%)	135 (47.5%)	0.251
Race, white, N(%)	195 (78.3%)	188 (66.2%)	0.005
Admitted from rehab or nursing facility, N(%)	25 (10.0%)	42 (14.8%)	0.099
BMI, kg/m^2, median (IQR)	26.9 (22.7, 32.4)	26.6 (22.5, 30.8)	0.360
Charlson comorbidity index, median (IQR)	4 (2,6)	4 (2,6)	0.804
Co-morbidities, N(%)			
Hypertension,	112 (45.0%)	141 (49.7%)	0.346
Diabetes	83 (33.3%)	87 (30.6%)	0.525
Malignancy$^{\perp}$	63 (25.3%)	56 (19.7%)	0.123
COPD	52 (20.9%)	43 (15.1%)	0.149
Congestive heart failure	33 (13.3%)	43 (15.1%)	0.526
Kidney disease (moderate/severe)	30 (12.1%)	39 (13.7%)	0.540
Peripheral vascular disease	21 (8.4%)	28 (9.9%)	0.544
Liver disease (moderate/severe)	15 (6.0%)	19 (6.7%)	0.758
Study arm, fluid-restrictive, N(%)	158 (63.5%)	181 (63.7%)	0.947
Enrolled in ED, N(%)	229 (92.0%)	255 (89.8%)	0.371
**Baseline* vitals and labs**			
MAP, mmHg, median (IQR)	67 (62, 73)	66 (61, 73)	0.225
Heart rate, beats per minute, median (IQR)	92 (80, 104)	96 (83, 110)	0.006
Respiratory rate, breaths per minute, median (IQR)	19 (16, 23)	20 (17, 24)	0.025
Glascow Coma Score, median (IQR)	15 (15, 15)	15 (14, 15)	0.006
Lactate, mmol/L, median (IQR)	2.1 (1.4, 3.4)	2.9 (1.8, 4.4)	<0.001
Creatinine, mg/dL, median (IQR)	1.5 (1.0, 2.3)	1.6 (1.0, 2.8)	0.157
On invasive ventilation, N(%)	12 (4.8%)	34 (12.0%)	0.003
On respiratory support**, N(%)	26 (10.4%)	54 (19.0%)	0.006
SOFA score, median (IQR)	4 (2, 6)	5 (3, 7)	0.009
**Management practices**			
Time to vasopressor initiation from hospital arrival in hours, median (IQR)	4.4 (2.7, 7.8)	4.7 (2.8, 7.8)	0.819
First vasopressor norepinephrine, N(%)	239 (96.0%)	268 (94.4%)	0.567
Peak norepinephrine dose (mcg/kg/min), day 1, median (IQR)	0.08 (0.05, 0.14)	0.16 (0.08, 0.28)	<0.001
Received a second vasopressor on day 1, N(%)	13 (5.2%)	71 (25.0%)	<0.001
On vasopressor beyond 24 hours, N(%)	135 (54.7%)	231 (81.3%)	<0.001
Total fluids in 24 hours, mL$^+$, median (IQR)	2250 (730, 5700)	4020 (1870, 6850)	<0.001
ICU admission on day 1, N(%)	221 (88.8%)	247 (87.0%)	0.210

eTable 10 Legend: This table compares baseline patient characteristics and management practices among patients who were alive at 72 hours (N=533) and received central access by 72 hours vs those who did not (received only peripheral vasopressors in the first 72 hours). Data are presented as median (IQR) or N (%). P-values were calculated using chi-squared test for categorical variables Mann Whitney U for continuous variables.

© 2025 Munroe ES et al. *JAMA Network Open*.

⊥ Malignancy includes solid tumor with or without metastasis, leukemia, and malignant lymphoma.
*Baseline vital signs and labs were the values recorded at the time of randomization
**Respiratory support includes mechanical ventilation, high flow nasal oxygen or non-invasive positive pressure ventilation. Excludes patients on chronic home mechanical ventilation.
+Total fluid from randomization to 24 hours, including crystalloid fluid boluses, albumin, maintenance fluid, blood product, and IV medication.
Definitions: IQR= interquartile range, BMI= body mass index, COPD= chronic obstructive pulmonary disease, ED= emergency department, MAP= mean arterial pressure, ICU= intensive care unit, SOFA= sequential organ failure assessment

© 2025 Munroe ES et al. *JAMA Network Open*.
