# Supplementary material for: Peripheral Vasopressor Use in Early Sepsis-Induced Hypotension
Source: JAMA Netw Open. 2025 Aug 27;8(8):e2529148. doi: 10.1001/jamanetworkopen.2025.29148 (PMC12391982; doi:10.1001/jamanetworkopen.2025.29148)
Supplement: Supplement 2. — Nonauthor Collaborators [file jamanetwopen-e2529148-s002.pdf]

Supplemental Online Content: Nonauthor Collaborators
*First name, last name, and suffix (if applicable) are required and will appear in PubMed.

*Group Name(s): The NHLBI PETAL Network							
***First Name and Middle Initial(s)**	***Last Name**	***Suffix (eg, Jr, III)**	Academic Degrees	Institution	Location (city, state/province, country)	Role or Contribution, eg, chair, principal investigator	Group (if more than 1 Group listed in the byline) and/or Subgroup (eg, Steering Committee)
Jay S	Steingrub			Baystate Medical Center		Clinical Center Principal Investigator	
Howard	Smithline			Baystate Medical Center			
Mark	Tidswell			Baystate Medical Center			
Lori	Kozikowski			Baystate Medical Center			
Sherell	Thornton-Thompson			Baystate Medical Center			
Lesley	De Souza			Baystate Medical Center			
Cynthia	Kardos			Baystate Medical Center			
Sarah	Romain			Baystate Medical Center			
Scott	Oullette			Baystate Medical Center			
Peter	Hou			Brigham and Women's Hospital		Clinical Center Principal Investigator	
Rebecca M	Baron			Brigham and Women's Hospital			
Anthony F	Massaro			Brigham and Women's Hospital			
Imoigele P	Aisiku			Brigham and Women's Hospital			
Raghu R	Seethala			Brigham and Women's Hospital			
Lauren N	Precopio			Brigham and Women's Hospital			
Torben K	Becker			University of Florida			
Christa M	Campbell			University of Florida			
Elida	Benitez			University of Florida			
Marie-Carmelle	Elie			University of Florida			
Matthew	Shaw			University of Florida			
Tori X	Tran			University of Florida			
Charles R	Wira	III		Yale New Haven Hospital			
Carolyn	Brokowski			Yale New Haven Hospital			
Jonathan M	Siner			Yale New Haven Hospital			
Justin B	Belsky			Yale New Haven Hospital			
Lauren E	Ferrante			Yale New Haven Hospital			

Supplemental Online Content: Nonauthor Collaborators
*First name, last name, and suffix (if applicable) are required and will appear in PubMed.

***First Name and Middle Initial(s)**	***Last Name**	***Suffix (eg, Jr, III)**	Academic Degrees	Institution	Location (city, state/province, country)	Role or Contribution, eg, chair, principal investigator	Group (if more than 1 Group listed in the byline) and/or Subgroup (eg, Steering Committee)
John E	Sather			Yale New Haven Hospital			
Ani	Aydin			Yale New Haven Hospital			
Nathan I	Shapiro			Beth Israel Deaconess Medical Center		Clinical Center Principal Investigator	
Daniel	Talmor			Beth Israel Deaconess Medical Center		Clinical Center Principal Investigator	
Valerie	Banner-Goodspeed			Beth Israel Deaconess Medical Center			
Thomas	O’Mara			Beth Israel Deaconess Medical Center			
Charlotte	Kirk			Beth Israel Deaconess Medical Center			
Kelly	Harrison			Beth Israel Deaconess Medical Center			
Lisa	Kurt			Beth Israel Deaconess Medical Center			
Margaret	Schermerhorn			Beth Israel Deaconess Medical Center			
Katherine	Boyle			Beth Israel Deaconess Medical Center			
Nicole	Dubosh			Beth Israel Deaconess Medical Center			
Sharon	Hayes			Beth Israel Deaconess Medical Center			
Eric	Hyder			Beth Israel Deaconess Medical Center			
David	Chiu			Beth Israel Deaconess Medical Center			
Oren	Mechanic			Beth Israel Deaconess Medical Center			

Supplemental Online Content: Nonauthor Collaborators
*First name, last name, and suffix (if applicable) are required and will appear in PubMed.

***First Name and Middle Initial(s)**	***Last Name**	***Suffix (eg, Jr, III)**	Academic Degrees	Institution	Location (city, state/province, country)	Role or Contribution, eg, chair, principal investigator	Group (if more than 1 Group listed in the byline) and/or Subgroup (eg, Steering Committee)
Ted	Raddell			Beth Israel Deaconess Medical Center			
Lihini	Keenawinna			Beth Israel Deaconess Medical Center			
Kostas	Andreo			Beth Israel Deaconess Medical Center			
Michael R	Filbin			Massachusetts General Hospital			
Kathryn	Hibbert			Massachusetts General Hospital			
Blair Alden	Parry			Massachusetts General Hospital			
Kendall	Lavin-Parsons			Massachusetts General Hospital			
Natalie	Pulido			Massachusetts General Hospital			
Alan E	Jones			University of Mississippi Medical Center			
James	Galbraith			University of Mississippi Medical Center			
Rebekah	Peacock			University of Mississippi Medical Center			
Ustav	Nandi			University of Mississippi Medical Center			
Michael A	Puskarich			Hennepin County Medical Center			
Matthew E	Prekker			Hennepin County Medical Center			
Audrey	Hendrickson			Hennepin County Medical Center			
Jamie	Stang			Hennepin County Medical Center			
Paige	DeVries			Hennepin County Medical Center			
David	Miru			St. Vincent Hospital			
Andre	de Souza Licht			St. Vincent Hospital			
Pam	Sigel			St. Vincent Hospital			
Patricia	Arsenault			St. Vincent Hospital			
Ronald A	Reilkoff			University of Minnesota Medical Center			

*First name, last name, and suffix (if applicable) are required and will appear in PubMed.

*First Name and Middle Initial(s)	*Last Name	*Suffix (eg, Jr, III)	Academic Degrees	Institution	Location (city, state/province, country)	Role or Contribution, eg, chair, principal investigator	Group (if more than 1 Group listed in the byline) and/or Subgroup (eg, Steering Committee)
Eric	Jaton			University of Minnesota Medical Center			
Abbey	Staugaitis			University of Minnesota Medical Center			
Michael	Matthay			UCSF San Francisco		Clinical Center Principal Investigator	
Kimberly	Yee			UCSF San Francisco			
Kimia	Ashktorab			UCSF San Francisco			
Anika	Agrawal			UCSF San Francisco			
Rachel	Gropper			UCSF San Francisco			
Steven Y	Chang			UCLA			
Gregory W	Hendey			UCLA		Clinical Center Principal Investigator	
George	Lim			UCLA			
Nida	Qadir			UCLA			
Andrea	Tam			UCLA			
Rebecca	Beutler			UCLA			
Joseph E	Levitt			Stanford University Hospital			
Jenny G	Wilson			Stanford University Hospital			
Angela J	Rogers			Stanford University Hospital			
Rosemary	Vojnik			Stanford University Hospital			
Jonasel	Roque			Stanford University Hospital			
Timothy E	Albertson			UC Davis			
James A	Chenoweth			UC Davis			
Christian	Sandrock			UC Davis			
Skyler J	Pearson			UC Davis			
Erin	Hardy			UC Davis			
Alyssa	Hughes			UCSF Fresno			
Kyndra	Sousa			UCSF Fresno			
Kinsley	Hubel			UCSF Fresno			
Eyad	Almarsi			UCSF Fresno			

Supplemental Online Content: Nonauthor Collaborators
*First name, last name, and suffix (if applicable) are required and will appear in PubMed.

***First Name and Middle Initial(s)**	***Last Name**	***Suffix (eg, Jr, III)**	Academic Degrees	Institution	Location (city, state/province, country)	Role or Contribution, eg, chair, principal investigator	Group (if more than 1 Group listed in the byline) and/or Subgroup (eg, Steering Committee)
Elizabeth	Vidales			University of Texas			
Bela	Patel			University of Texas			
Ryan	Huebinger			University of Texas			
Adit A	Ginde			University of Colorado Hospital		Clinical Center Principal Investigator	
Marc	Moss			University of Colorado Hospital		Clinical Center Principal Investigator	
Neil	Aggarwal			University of Colorado Hospital			
Jeffrey	McKeehan			University of Colorado Hospital			
Lani	Finck			University of Colorado Hospital			
Carrie	Higgins			University of Colorado Hospital			
Michelle	Howell			University of Colorado Hospital			
Ivor S	Douglas			Denver Health Medical Center			
Jason	Haukoos			Denver Health Medical Center			
Stacy	Trent			Denver Health Medical Center			
Terra	Hiller			Denver Health Medical Center			
Carolynn	Lyle			Denver Health Medical Center			
Ana	Garcia			Denver Health Medical Center			
Stephanie	Gravitz			Denver Health Medical Center			
Ivan N	Co			University of Michigan			
Pauline K	Park			University of Michigan		Clinical Center Principal Investigator	
Robert	Hyzy			University of Michigan		Clinical Center Principal Investigator	
Kristine	Nelson			University of Michigan			
J Victor	Jimenez			University of Michigan			
Christopher M-C	Fung			University of Michigan			
Jakob I	McSparron			University of Michigan			
Norman	Olbrich			University of Michigan			
Sinan	Hanna			University of Michigan			
Mark	Williams			Indiana University			

Supplemental Online Content: Nonauthor Collaborators
*First name, last name, and suffix (if applicable) are required and will appear in PubMed.

*First Name and Middle Initial(s)	*Last Name	*Suffix (eg, Jr, III)	Academic Degrees	Institution	Location (city, state/province, country)	Role or Contribution, eg, chair, principal investigator	Group (if more than 1 Group listed in the byline) and/or Subgroup (eg, Steering Committee)
Raj	Kapoor			Indiana University			
Jean	Nash			Indiana University			
Meghan	Willig			Indiana University			
Robert	Sherwin			Wayne State University			
Robert	Ehrman			Wayne State University			
James	Paxton			Wayne State University			
John	Wilburn			Wayne State University			
Michelle Ng	Gong			Montefiore Moses		Clinical Center Principal Investigator	
Ari	Moskowitz			Montefiore Moses			
Rahul	Nair			Montefiore Moses			
William	Nkemdirim			Montefiore Moses			
Hiwet	Tzehaie			Montefiore Moses			
Jen-Ting	Chen			Montefiore Weiler			
Amira	Mohamed			Montefiore Weiler			
Brenda	Lopez			Montefiore Weiler			
Sabah	Boujid			Montefiore Weiler			
Manuel Hache	Marliere			Montefiore Weiler			
Lynne D	Richardson			Mt. Sinai Hospital		Clinical Center Principal Investigator	
Kusum	Mathews			Mt. Sinai Hospital			
Patrick	Maher			Mt. Sinai Hospital			
Samuel	Acquah			Mt. Sinai Hospital			
Neha	Goel			Mt. Sinai Hospital			
Jarrod M	Mosier			University of Arizona			
Cameron	Hypes			University of Arizona			
Bhupinder	Natt			University of Arizona			
Bryan A	Borg			University of Arizona			
Elizabeth Salvagio	Campbell			University of Arizona			
Kristin M	Hudock			University of Cincinnati Medical Center			

*First name, last name, and suffix (if applicable) are required and will appear in PubMed.

***First Name and Middle Initial(s)**	***Last Name**	***Suffix (eg, Jr, III)**	Academic Degrees	Institution	Location (city, state/province, country)	Role or Contribution, eg, chair, principal investigator	Group (if more than 1 Group listed in the byline) and/or Subgroup (eg, Steering Committee)
Opeolu	Adeoye			University of Cincinnati Medical Center			
R. Duncan	Hite			University of Cincinnati Medical Center			
Evan L	Ramser			University of Cincinnati Medical Center			
Michael	Hellman			University of Cincinnati Medical Center			
Autumn	Cresie			University of Cincinnati Medical Center			
Sara	Keegan			University of Cincinnati Medical Center			
Abhijit	Duggal			Cleveland Clinic Foundation		Clinical Center Principal Investigator	
Siddharth	Dugar			Cleveland Clinic Foundation			
Omar	Mehkri			Cleveland Clinic Foundation			
Andrei	Hastings			Cleveland Clinic Foundation			
Kiran	Ashok			Cleveland Clinic Foundation			
Stephanie	Stoianoff			Cleveland Clinic Foundation			
Matthew C	Exline			Ohio State University Wexner Medical Center			
Jason J	Bischof			Ohio State University Wexner Medical Center			
Thomas E	Terndrup			Ohio State University Wexner Medical Center			
Henry E	Wang			Ohio State University Wexner Medical Center			
Joshua A	Englert			Ohio State University Wexner Medical Center			
Jennifer A	Frey			Ohio State University Wexner Medical Center			

Supplemental Online Content: Nonauthor Collaborators
*First name, last name, and suffix (if applicable) are required and will appear in PubMed.

***First Name and Middle Initial(s)**	***Last Name**	***Suffix (eg, Jr, III)**	Academic Degrees	Institution	Location (city, state/province, country)	Role or Contribution, eg, chair, principal investigator	Group (if more than 1 Group listed in the byline) and/or Subgroup (eg, Steering Committee)
Sarah C	Karow			Ohio State University Wexner Medical Center			
D. Mark	Courtney			Northwestern University			
Richard	Wunderink			Northwestern University			
Helen	Donnelly			Northwestern University			
Megan	Rowland			Northwestern University			
Kate	Piserchia			Northwestern University			
Nicholas J	Johnson			Harborview Medical Center			
Bryce RH	Robinson			Harborview Medical Center		Clinical Center Principal Investigator	
Catherine L	Hough			Harborview Medical Center		Clinical Center Principal Investigator	
Stephanie	Gundel			Harborview Medical Center			
Sakshi	Seghal			Harborview Medical Center			
Sarah	Katsandres			Harborview Medical Center			
Sarah	Dean			Harborview Medical Center			
Kelsey	Jiang			Harborview Medical Center			
Megan	Fuentes			Harborview Medical Center			
Maranda	Newton			Harborview Medical Center			
Emily	Petersen			Harborview Medical Center			
Akram	Khan			Oregon Health and Science University			
Olivia	Krol			Oregon Health and Science University			
Milad Karami	Jouzestani			Oregon Health and Science University			
Makrina	Kamel			Oregon Health and Science University			
Ebaad	Haq			Oregon Health and Science University			

Supplemental Online Content: Nonauthor Collaborators
*First name, last name, and suffix (if applicable) are required and will appear in PubMed.

***First Name and Middle Initial(s)**	***Last Name**	***Suffix (eg, Jr, III)**	Academic Degrees	Institution	Location (city, state/province, country)	Role or Contribution, eg, chair, principal investigator	Group (if more than 1 Group listed in the byline) and/or Subgroup (eg, Steering Committee)
Zach	Zouyed			Oregon Health and Science University			
Peter	Chen			Cedars Sinai Medical Center			
Sam	Torbati			Cedars Sinai Medical Center			
Susan	Jackman			Cedars Sinai Medical Center			
Niree	Hindoyan			Cedars Sinai Medical Center			
Joseph	Meza			Cedars Sinai Medical Center			
Shane	O'Mahony			Swedish Hospital First Hill			
Julie	Wallick			Swedish Hospital First Hill			
Alexandra	Duven			Swedish Hospital First Hill			
Dakota	Fletcher			Swedish Hospital First Hill			
Alexandra	Weissman			UPMC Presbyterian, Shadyside & Mercy			
Donald M	Yealy			UPMC Presbyterian, Shadyside & Mercy		Clinical Center Principal Invesitgator	
Sarah	McGarry			UPMC Presbyterian, Shadyside & Mercy			
Bryan J	McVerry			UPMC Presbyterian, Shadyside & Mercy			
David T	Huang			UPMC Presbyterian, Shadyside & Mercy			
Michael A	Turturro			UPMC Presbyterian, Shadyside & Mercy			
Derek C	Angus			UPMC Presbyterian, Shadyside & Mercy		Clinical Center Principal Invesitgator	
Jordan	Schooler			Penn State Hershey Medical Center			
Lawrence E	Kass			Penn State Hershey Medical Center			
Nina T	Gentile			Temple University Hospital			
Nathaniel	Marchetti			Temple University Hospital			

Supplemental Online Content: Nonauthor Collaborators
*First name, last name, and suffix (if applicable) are required and will appear in PubMed.

***First Name and Middle Initial(s)**	***Last Name**	***Suffix (eg, Jr, III)**	Academic Degrees	Institution	Location (city, state/province, country)	Role or Contribution, eg, chair, principal investigator	Group (if more than 1 Group listed in the byline) and/or Subgroup (eg, Steering Committee)
Hannah	Reimer			Temple University Hospital			
D. Clark	Files			Wake Forest Baptist Health		Clinical Center Principal Invesitgator	
Kevin W	Gibbs			Wake Forest Baptist Health			
Chadwick	Miller			Wake Forest Baptist Health		Clinical Center Principal Invesitgator	
Mary	LaRose			Wake Forest Baptist Health			
Lori	Flores			Wake Forest Baptist Health			
Lauren	Koehler			Wake Forest Baptist Health			
Peter E	Morris			University of Kentucky			
Jamie	Sturgill			University of Kentucky			
Ashley	Montgomery-Yates			University of Kentucky			
Evan P	Cassity			University of Kentucky			
Sanjay	Dhar			University of Kentucky			
Marjolein	de Wit			Virginia Commonwealth University Medical Center			
Jessica	Mason			Virginia Commonwealth University Medical Center			
Aamer	Syed			Virginia Commonwealth University Medical Center			
Andrew J	Goodwin			Medical University of South Carolina			
Abbey	Grady			Medical University of South Carolina			
Caitlan	Lematty			Medical University of South Carolina			
Charles	Terry			Medical University of South Carolina			
Cynthia	Oliva			Medical University of South Carolina			

Supplemental Online Content: Nonauthor Collaborators
*First name, last name, and suffix (if applicable) are required and will appear in PubMed.

***First Name and Middle Initial(s)**	***Last Name**	***Suffix (eg, Jr, III)**	Academic Degrees	Institution	Location (city, state/province, country)	Role or Contribution, eg, chair, principal investigator	Group (if more than 1 Group listed in the byline) and/or Subgroup (eg, Steering Committee)
Kyle	Enfield			University of Virginia Medical Center			
Mark	Sochor			University of Virginia Medical Center			
Mary	Marshall			University of Virginia Medical Center			
Miranda	West			University of Virginia Medical Center			
Ashley	Simpson			University of Virginia Medical Center			
Lindsay M	Leither			Intermountain Medical Center			
Samuel M	Brown			Intermountain Medical Center		Clinical Center Principal Invesitgator	
Ithan	Peltan			Intermountain Medical Center			
Joseph	Bledsoe			Intermountain Medical Center		Clinical Center Principal Invesitgator	
Melissa	Fergus			Intermountain Medical Center			
Valerie	Aston			Intermountain Medical Center			
Quinn	Montgomery			Intermountain Medical Center			
Rilee	Smith			Intermountain Medical Center			
Katie	Brown			Intermountain Medical Center			
Brent	Armbruster			Intermountain Medical Center			
Darrin	Applegate			Intermountain Medical Center			
Estelle	Harris			University of Utah Hospital			
Elizabeth A	Middleton			University of Utah Hospital			
Robert	Paine	III		University of Utah Hospital			
Lindsey J	Waddoups			University of Utah Hospital			
Amber	Plante			University of Utah Hospital			
Scott	Youngquist			University of Utah Hospital			
John	Eppensteiner			Duke University Medical Center			
Andrew	Bouffler			Duke University Medical Center			

*First name, last name, and suffix (if applicable) are required and will appear in PubMed.

***First Name and Middle Initial(s)**	***Last Name**	***Suffix (eg, Jr, III)**	Academic Degrees	Institution	Location (city, state/province, country)	Role or Contribution, eg, chair, principal investigator	Group (if more than 1 Group listed in the byline) and/or Subgroup (eg, Steering Committee)
Christopher	Cox			Duke University Medical Center			
Alexander T	Limkakeng			Duke University Medical Center			
Adam	Breslin			Duke University Medical Center			
Bennett	deBoisblanc			Louisiana State University Health Sciences Center			
Matthew	Lammi			Louisiana State University Health Sciences Center			
Kyle	Happel			Louisiana State University Health Sciences Center			
David	Janz			Louisiana State University Health Sciences Center			
Paula	Lauto			Louisiana State University Health Sciences Center			
Connie	Romaine			Louisiana State University Health Sciences Center			
Marie Childs	Sandi			Louisiana State University Health Sciences Center			
Jonathan D	Casey			Vanderbilt University Medical Center			
Margaret	Hays			Vanderbilt University Medical Center			
Adrienne	Baughman			Vanderbilt University Medical Center			
Shannon	Pugh			Vanderbilt University Medical Center			
Jakea	Johnson			Vanderbilt University Medical Center			
David B	Page			University of Alabama Birmingham			
Derek W	Russell			University of Alabama Birmingham			

Supplemental Online Content: Nonauthor Collaborators
*First name, last name, and suffix (if applicable) are required and will appear in PubMed.

***First Name and Middle Initial(s)**	***Last Name**	***Suffix (eg, Jr, III)**	Academic Degrees	Institution	Location (city, state/province, country)	Role or Contribution, eg, chair, principal investigator	Group (if more than 1 Group listed in the byline) and/or Subgroup (eg, Steering Committee)
Donna S	Harris			University of Alabama Birmingham			
Shannon S	Carson			University of North Carolina at Chapel Hill			
Jason	Mock			University of North Carolina at Chapel Hill			
Eugenia B	Quackenbush			University of North Carolina at Chapel Hill			
Colleen	Rice			University of North Carolina at Chapel Hill			
David A	Schoenfeld			Massachusetts General Hospital Biostatistics Center (CCC)		CCC PI	
B. Taylor	Thomspon			Massachusetts General Hospital Biostatistics Center (CCC)		CCC PI	
Douglas L	Hayden			Massachusetts General Hospital Biostatistics Center (CCC)			
Nancy	Ringwood			Massachusetts General Hospital Biostatistics Center (CCC)			
Cathryn	Oldmixon			Massachusetts General Hospital Biostatistics Center (CCC)			
Christine	Ulysse			Massachusetts General Hospital Biostatistics Center (CCC)			
Richard	Morse			Massachusetts General Hospital Biostatistics Center (CCC)			
Ariela	Muzikansky			Massachusetts General Hospital Biostatistics Center (CCC)			
Laura	Fitzgerald			Massachusetts General Hospital Biostatistics Center (CCC)			
Samuel	Whitaker			Massachusetts General Hospital Biostatistics Center (CCC)			

Supplemental Online Content: Nonauthor Collaborators
*First name, last name, and suffix (if applicable) are required and will appear in PubMed.

***First Name and Middle Initial(s)**	***Last Name**	***Suffix (eg, Jr, III)**	Academic Degrees	Institution	Location (city, state/province, country)	Role or Contribution, eg, chair, principal investigator	Group (if more than 1 Group listed in the byline) and/or Subgroup (eg, Steering Committee)
Adrian	Lagakos			Massachusetts General Hospital Biostatistics Center (CCC)			
Weixing	Huang			Massachusetts General Hospital Biostatistics Center (CCC)			
Poying	Lai			Massachusetts General Hospital Biostatistics Center (CCC)			
Hayley	Morin			Massachusetts General Hospital Biostatistics Center (CCC)			
Roy G.	Brower			Johns Hopkins University		Steering Committee Chair	
Lora A	Reineck			National Heart, Lung, and Blood Institute			
Karen	Bienstock			National Heart, Lung, and Blood Institute			
Ejigayehu	Demissie			National Heart, Lung, and Blood Institute			
Michelle	Freemer			National Heart, Lung, and Blood Institute			
James	Kiley			National Heart, Lung, and Blood Institute			
Lauren	Kunz			National Heart, Lung, and Blood Institute			
Mario	Stylianou			National Heart, Lung, and Blood Institute			
Myron	Maclawiw			National Heart, Lung, and Blood Institute			
Gail	Weinmann			National Heart, Lung, and Blood Institute			
Laurie J	Morrison					Protocol Review Committee	

Supplemental Online Content: Nonauthor Collaborators
*First name, last name, and suffix (if applicable) are required and will appear in PubMed.

***First Name and Middle Initial(s)**	***Last Name**	***Suffix (eg, Jr, III)**	Academic Degrees	Institution	Location (city, state/province, country)	Role or Contribution, eg, chair, principal investigator	Group (if more than 1 Group listed in the byline) and/or Subgroup (eg, Steering Committee)
Daniel	Brodie					Protocol Review Committee	
Charles B	Cairns					Protocol Review Committee	
Mark N	Gillespie					Protocol Review Committee	
Richard J	Kryscio					Protocol Review Committee	
Damon	Scales					Protocol Review Committee	
Polly	Parsons					Data And Safety Monitoring Board	
Jason D	Christie					Data And Safety Monitoring Board	
Neal	Dickert	Jr				Data And Safety Monitoring Board	
Deborah	Diercks					Data And Safety Monitoring Board	
Jesse R	Hall					Data And Safety Monitoring Board	
Nicholas J	Horton					Data And Safety Monitoring Board	
Mitchell	Levy					Data And Safety Monitoring Board	
Mark	Siegel					Data And Safety Monitoring Board	
Ian	Stiell					Data And Safety Monitoring Board	
Laurie S	Zoloth					Data And Safety Monitoring Board	
