# Supplementary material for: Peripheral Vasopressor Use in Early Sepsis-Induced Hypotension
Source: JAMA Netw Open. 2025 Aug 27;8(8):e2529148. doi: 10.1001/jamanetworkopen.2025.29148 (PMC12391982; doi:10.1001/jamanetworkopen.2025.29148)
Supplement: Supplement 3. — Data Sharing Statement [file jamanetwopen-e2529148-s003.pdf]

Data Sharing Statement

Munroe. Peripheral Vasopressor Use in Early Sepsis-Induced Hypotension. *JAMA Netw Open*. Published August 27, 2025. doi:10.1001/jamanetworkopen.2025.29148

Data
Data available: No

Additional Information
Explanation for why data not available: Data from the CLOVERS trial are publicly available on BioLINCC
